# On-target and direct modulation of alloreactive T cells by a nanoparticle carrying MHC alloantigen, regulatory molecules and CD47 in a murine model of alloskin transplantation

**DOI:** 10.1080/10717544.2018.1447049

**Published:** 2018-03-06

**Authors:** Khawar Ali Shahzad, Xin Wan, Lei Zhang, Weiya Pei, Aifeng Zhang, Muhammad Younis, Wei Wang, Chuanlai Shen

**Affiliations:** a Department of Microbiology and Immunology, Medical School, Southeast University, Nanjing, Jiangsu, China;; b Department of Pathology, Medical School, Southeast University, Nanjing, Jiangsu, China;; c State Education Ministry’s Key Laboratory of Development Genes and Human Disease, Institute of Life Sciences, Southeast University, Nanjing, Jiangsu, China

**Keywords:** Alloskin transplantation, alloreactive T cells, major histocompatibility complex, biomimetic nanoparticles, immunotherapy

## Abstract

Biomimetic nanoparticles have been reported as immune modulators in autoimmune diseases and allograft rejections by numerous researchers. However, most of the therapeutics carrying antigens, toxins or cytokines underlay the mechanism of antigen presentation by cellular uptake of NPs through pinocytosis and phagocytosis. Few researches focus on the direct and antigen-specific modulation on T cells by NPs and combined use of multiple regulatory molecules. Here, polylactic-co-glycolic acid nanoparticles (PLGA-NPs) were fabricated as scaffold to cocoupling H-2K^b^-Ig dimer, anti-Fas mAb, PD-L1-Fc, TGF-β and CD47-Fc for the generation of alloantigen-presenting and tolerance-inducing NPs, termed killer NPs and followed by *i.v.* injection into a single MHC-mismatched murine model of alloskin transplantation. Three infusions prolonged alloskin graft survival for 45 days; depleted most of H-2K^b^ alloreactive CD8^+^ T cells in peripheral blood, spleen and local graft, in an antigen-specific manner. The killer NPs circulated throughout vasculature into various organs and local allograft, with a retention time up to 30 h. They made contacts with CD8^+^ T cells to facilitate vigorous apoptosis, inhibit the activation and proliferation of alloreactive CD8^+^ T cells and induce regulatory T cells in secondary lymphoid organs, with the greatly minimized uptake by phagocytes. More importantly, the impairment of host overall immune function and visible organ toxicity were not found. Our results provide the first experimental evidence for the direct and on-target modulation on alloreactive T cells by the biodegradable 200-nm killer NPs via co-presentation of alloantigen and multiple regulatory molecules, thus suggest a novel antigen-specific immune modulator for allograft rejections.

## Introduction

In the past 20 years, allograft transplantation has enabled the recipients to enjoy promising transplanted graft survival (Page et al., 2012), but the long-term clinical outcomes (up to 10 years) depend on the continuous usage of immunosuppressive agents (Angaswamy et al., 2013), which often results in nonspecific suppression of overall host immune system and make the host body more prone to different simple infections (Masri, 2003). The holy grail for the treatment of allo-rejection is to modulate the immune system by inducing antigen-specific tolerance which should not impair the overall immune function (Fisher et al., 2015). The progress of micro- and nanotechnology facilitates the researches using biomimetic particles as immune modulators (Raich-Regue et al., 2014). Killer and artificial antigen-presenting cell (KaAPC) is one of the fundamental strategies. The peptide-major histocompatibility complex (pMHC) multimers and anti-Fas mAb were covalently co-coupled on the surface of cell-sized polymer particles to directly induce the apoptosis of antigen-specific T cells in static 96-well plates (Schutz et al., 2008). In our recent works, the polylactic-co-glycolic acid (PLGA), a biocompatible and biodegradable polymer, was used to generate cell-sized microparticles (PLGA-MPs) to co-coupling H-2K^b^-Ig dimer and anti-Fas mAb. The biodegradable KaAPCs or killer PLGA-MPs could directly deplete OVA_257–264_-specific CD8^+^ T cells in an OVA antigen-specific manner and Fas/FasL-dependent fashion, both *in vitro* and in OT-1 mice (Wang et al., 2016), and also markedly prolonged the alloskin graft survival in a murine model by selectively depleting the H-2K^b^-alloreactive CD8^+^ T cells after intravenous injections (Wang et al., 2017). However, despite the encouraging results and prospects, the use of cell-sized PLGA-MPs as an acellular scaffold may evoke concerns regarding biosafety and organ toxicity for the putative clinical use. Large-sized MPs can cause the clinical problems, such as hindering blood flow by lodging the pulmonary vasculature, accumulating in terminal organs, and resulting in stroke in the recipients when intravenous (*i.v.*) injection (Champion et al., 2008). Another limitation is the failure of local delivery at the site of interest since their weak ability to cross most biological barriers (Glowacki et al., 2015).

Nanoparticles (NPs) are found an excellent substitute for MPs as carrier platform of drug and vaccine delivery. Because of too small size, NPs can cross the biological barriers easily, circulate without hindering blood flow and do not accumulate in terminal organs (Harris et al., 2001). Therefore, a nanoscale KaAPC should be further investigated. PLGA-NPs are approved by the United States Food and Drug Administration as safe to use in human in different diagnostic and therapeutic applications (Lu et al., 2009; Danhier et al., 2012; Ye et al., 2014). In the present study, polyethyleneimine (PEI)-coated PLGA-NPs were fabricated as a nanoscale scaffold to covalently co-coupling targeting antigen (H-2K^b^-Ig dimer), modulators (anti-Fas mAb, PD-L1-Fc, TGF-β) and ‘self-marker’ (CD47-Fc) for the direct and on-target depletion and modulation of alloreactive T cells. The killer PLGA-NPs were administered *i.v.* into bm1 mice (H-2K^bm1^) that had previously been grafted with ear skin from C57BL/6 mice (H-2K^b^), a single MHC-mismatched murine model of alloskin transplantation, followed by the investigation of therapeutic outcome, precise mechanism, tissue distribution, side effects and organ toxicity. The intriguing results highlight, for the first time, the therapeutic capability of the killer NPs to directly modulate alloreactive T cells for the treatment of allograft rejections.

## Materials and methods

### Mice and cell lines

The bm1 mice (B6.C-H2^bm1/ByJ^) were purchased from the Jackson Laboratory (Bar Harbor, CA, USA) and bred in-house. Male C57BL/6 (H-2K^b^) and BALB/c (H-2K^d^) mice were acquired from the Comparative Medicine Center, Yangzhou University (Yangzhou, Jiangsu, China). All mice were maintained in the specific pathogen-free laboratory, Animal Center of Southeast University (Nanjing, Jiangsu, China) and were used in experiments at 8–9 weeks of age. All the animal welfare and experimental procedures were performed according to the protocols approved by Animal Ethics Committee of Southeast University and were consistent with the National Institutes of Health Guide for the Care and Use of Laboratory Animals (NIH Publications No. 8023, revised 1978). The melanoma B16F10 cell line was obtained from the Cell Bank of Type Culture Collection of Chinese Academy of Sciences (Shanghai, China).

### Preparation of PLGA-NPs and ICG-encapsulated PLGA-NPs

A double emulsion solvent evaporation method was used to prepare the PEI-coated PLGA-NPs and indocyanine green (ICG)-encapsulated PLGA-NPs as described by Meyer (Meyer et al., 2015b), with minor modifications. Briefly, 100 mg of PLGA (Daigang Co, Jinan, China) was added in 5 mL of dichloromethane with or without ICG (Sigma-Aldrich, St Louis, MO) and dissolved completely. The prepared solution was sonicated by microtip probe sonicator (Microson XL 2000, Misonix Inc., Farmingdale, NY) for 3 min, then added into 25 mL of 1% poly vinylalcohol (PVA) solution (Sigma-Aldrich) and sonicated again with various durations depending on the required size of PLGA-NPs. Finally, the resulting emulsification was mixed in 50 mL of 0.5% PVA solution. Dichloromethane was allowed to evaporate from the solution by magnetic stir bar agitation for 6 hr. The large-sized PLGA particles were removed from the solution by centrifugation at 4000 g for 5 min. The supernatant was collected and ultra-centrifuged three times at 17,000 *g* for 10 min/time to remove the PVA. The surface activation was carried out by mixing the prepared PLGA-NPs with 1-ethyl-3-(3-dimethylaminopropyl) carbodiimide hydrochloride (EDC) and *N*-hydroxysuccinimide (NHS) (Sigma-Aldrich). The solution was then added dropwise in PEI (Sigma-Aldrich) on a rotator with a magnetic stirrer at room temperature (RT, 25 °C) for 4 h. Finally, the excessive PEI was removed by wishing with deionized H_2_O.

The size and zeta potential of prepared NPs was measured using Zetasizer (Nano-ZS90, Malvern, England). The shape and surface morphology was observed under scanning electron microscope (SEM, Hitachi S-4800, Japan).

### Preparation of killer NPs, ICG-encapsulated killer NPs and PE-labeled killer NPs

The prepared approximately 80-nm or 200-nm PLGA-NPs (1 mg) were incubated with H-2K^b^-Ig dimer (10 μg, BD Biosciences, San Jose, CA), anti-Fas mAb (10 μg, Jo2, BD Biosciences), PD-L1-Fc (10 µg, Sino Biological Inc, Beijing, China), TGF-β (2.5 µg, PeproTech Inc, Rocky Hill, NJ), and CD47-Fc (0.3 µg, R&D Systems, Minneapolis, MN) in sterile 0.1 M PBS on a rotator at 4 °C for 24 hr. Then, blocking buffer (0.1 M PBS with 0.01% NaN_3_ and 10% mouse serum) was added to the tubes and incubated again for 24 h at 4 °C on a rotator. The resulting killer NPs were finally washed two times with sterile 0.1 M PBS before use. In parallel, several controls of the killer NPs were also prepared by following the similar procedure, such as NP^aFas^ (only coupling anti-Fas), NP^Kb^ (only coupling H-2K^b^-Ig dimer), NP^CD47^(only coupling CD47-Fc), NP^Kb/aFas^ (co-coupling H-2K^b^-Ig dimer and anti-Fas) and NP^Kb/aFas/PD-L1/TGFβ^ (co-coupling H-2K^b^-Ig dimer, anti-Fas, PD-L1-Fc and TGF-β). For the *in vivo* imaging of killer NPs, ICG-encapsulated killer NPs was prepared similarly by using ICG-encapsulated PLGA-NPs as a scaffold. R-phycoerythrin (PE)-labeled killer NPs were prepared by coincubating PE-streptavidin (BD Biosciences) (15 μg) and other immune molecules with PLGA-NPs in a similar way. Blank NPs were prepared by blocking PLGA-NPs with bovine serum albumin (BSA).

For phenotypic analyses, PE-anti-mouse H-2K^b^ mAb (AF6-88.5, BD Biosciences), FITC-anti-hamster IgG mAb (binding to anti-Fas mAb, G192-1, BD Biosciences), and APC-anti-human IgG1 (binding to PD-L1-Fc and CD47-Fc, Miltenyi Biotech, Bergisch Gladbach, Germany) were co-incubated with 1 mg of 80-nm or 200-nm killer NPs at 4 °C for 40 min with rotation and then blocked with 10% BSA in PBS for 12 h. After two times washing with PBS, the killer NPs were observed under confocal laser scanning microscopy (FV1000; Olympus Corporation, Tokyo, Japan).

### Skin transplantation and treatment with killer NPs

Skin transplantation was performed by following the procedure described by Garrod (Garrod & Cahalan, 2008) and Wang (Wang et al., 2017) with minor modifications. Briefly, the dorsal tissue from the ear of male C57BL/6 J was prepared (0.5-0.5 cm), and hair of male bm1 mice was removed from the dorsal flank area under anesthesia. Then, the prepared tissue was grafted, and a BAND AID® styptic plaster containing benzalkonium chloride (Shanghai Johnson &Johnson, Ltd., Shanghai, China) was placed over the grafted area for 5 days. The mice with skin graft were housed independently. The styptic plaster was then removed, and the bm1 mice with successful graft operation were assigned randomly to 1 of 8 groups and injected intravenously through tail vein with 200-nm Killer NPs, NP^aFas^, NP^Kb^, NP^CD47^, NP^Kb/aFas^, NP^/Kb/aFasPD-L1/TGFβ^, Blank NPs or sterile PBS on days 9, 11 and 13 after transplantation with 1 mg of NPs/mouse/time point. Same was the procedure followed for 80-nm killer NPs and its three control groups (NP^aFas/Kb/PD-L1/TGFβ^, Blank NPs and PBS). The rejection signs for allograft were monitored on daily basis. Grafts were defined as rejected when less than 10% of the graft remained viable.

### Immunofluorescence and histopathological analyses of alloskin graft

To evaluate the local infiltration of H-2K^b^-alloreactive T cells, CD4^+^ T cells and CD8^+^ T cells, immunofluorescence staining was performed on alloskin sections. On day 15 after skin transplantation, the full-thickness alloskin grafts were dissected from recipient bm1 mice of each group. The skin specimens were fixed in 4% formaldehyde and embedded in paraffin freezing medium (O.C.T, Sakura Finetek USA Inc, Torrance, CA). The sections with a thickness of 9–10 μm were prepared and followed by treatment with 3% H_2_O_2_ in methanol for 1 h and incubation with 10% mouse serum-PBS and anti-mouse CD16/CD32 (2.4G2, BD Biosciences) for 30 min at RT. The sections were then incubated with the mixture of H-2K^b^-Ig dimer and PE-labeled anti-mouse IgG1 (A85-1, BD Biosciences) at RT for 1 h. In parallel, the mouse IgG1 isotype control (eBiosciences) was also used to stain with the sections in the same manner. After washing with PBS, the sections were stained with DAPI (Sigma-Aldrich) for 5 min. After three times washing with PBS, the sections were imaged by using confocal laser scanning microscopy (Olympus).

Additionally, the frozen sections were incubated with rat anti-mouse CD8 (H35-17.2, eBiosciences, San Diego, CA), rat anti-mouse CD4 (GK1.5, eBiosciences) or rat IgG2b isotype control (eBiosciences) overnight at 4 °C and then incubated with biotinylated mouse anti-rat IgG (H + L) antibodies (eBiosciences) at RT for 1 h. The sections were visualized using the ABC kit (Boster Biological Technology, Ltd, Wuhan, China) for immunohistochemical analyses after washing two times with sterile PBS. For the assessment of inflammation, alloskin sections with a thickness of 6–7 μm were prepared using paraffin embedding technique and stained with hematoxylin and eosin (H&E) routinely. Each stained section was observed under light microscope (Nikon). The positive staining mean percentage was calculated by counting 10 different fields at 200 × magnification, using Image-Pro Plus software (Media Cybernetics, Rockville, MD).

### Detection of alloantigen-reactive CD8^+^ T cells

Peripheral blood and spleens were used to detect the frequency of H-2K^b^ alloantigen-reactive CD8^+^ T cells. On day 15 after skin transplantation, spleens and peripheral blood were collected from recipient bm1 and processed to cell suspensions. Firstly, anti-mouse CD16/CD32 (2.4G2, BD Biosciences) was used to block the cells (1 μg/8 × 10^5^ cells) for 30 min. Then, the cells were then incubated for 1 h with the mixture of APC-anti-mouse IgG1 and peptide-unloaded H-2K^b^-Ig dimer (BD Biosciences) at 4 °C for 1 h. The cells were washed twice and incubated again for 30 min with PE-anti-mouse CD3e (145-2C11, eBiosciences) and FITC-anti-mouse CD8a (53-6.7, eBiosciences). Then, cells were washed with sterile PBS and acquired on a FACS Calibur flow cytometer (BD Biosciences). The data were analyzed by using the flowJo software (Tree Star, Ashland, OR).

### Analyses of CD8^+^ T cells apoptosis and activation

On day 15, the splenocytes from recipient bm1 mice were prepared and incubated with APC-anti-mouse CD8a (53-6.7, eBiosciences) for 30 min at 4 °C and then stained with Annexin V and propidium iodide (PI) according to the manufacturer’s protocol (Dead Cell Apoptosis Kit, Invitrogen, Carlsbad, CA). To detect CD8^+^ T cells activation, the splenocytes were stained with FITC-anti-mouse CD3 (S4.1, eBiosciences), APC-anti-mouse CD8 (3B5, eBiosciences) and PE-anti-mouse CD44 (156-3C11, eBiosciences). The cells were then analyzed by using flow cytometry as described.

### T cell proliferation and anti-donor mixed lymphocyte reaction (MLR) assay

The recipient mice from each treatment group were sacrificed, and spleens were collected on day 15 after skin transplantation. Splenocytes were incubated with 5 μM carboxyfluorescein succinimidyl ester (CFSE, Sigma-Aldrich) at 37 °C for 10 min, and then immediately washed three times with ice-cold RPMI 1640 medium (Gibco BRL, Grand Island, NY, USA). The resulting CFSE-labeled spleen cells (1 × 10^5^ cells/well) were seeded as responder cells into 96-well round-bottom plate (BD Falcon) and coincubated with the stimulator cells (1 × 10^5^ cells/well, Mitomycin C-pretreated spleen cells from donor C57BL/6 J mice) in complete RPMI1640 medium (Gibco BRL) for 7 days at 37 °C, 5% CO_2_ and humidified conditions. After incubation, the collected cells were stained with APC-anti-mouse CD3e (145-2C11, eBiosciences) at 4 °C for 30 min, washed with sterile PBS and analyzed by Flow cytometry. 2 × 10^5^ events were collected for each sample. Cell divisions were demarcated according to CFSE-staining brightness. A third-party MLR was also performed as a control group in which Mitomycin C-pretreated spleen cells from BALB/c mice (H-2K^d^) rather than C57BL/6 J mice were cocultured with the responder cells in the same manner.

### Detection of regulatory CD4^+^ T cells

To detect the regulatory T cells, spleens and lymph nodes were collected on day 15 after transplantation from recipient bm1 mice in each group and processed to single cells suspensions. The protocol for Mouse Regulatory T Cell Staining Kit (eBiosciences) was followed. Briefly, the cell suspensions were blocked with anti-mouse CD16/CD32 (2.4G2) and then stained with APC-anti-mouse CD25 (PC61.5) and FITC-anti-mouse CD4 (RM4-5). After fixation, the intracellular staining with PE-anti-mouse Foxp3 (FJK-16 s) was performed and finally analyzed by flow cytometry.

### *In vivo* and *ex vivo* near-infrared imaging

ICG-encapsulated killer NPs, H-2K^b-^ killer NPs (H-2K^b^-Ig dimer absent), CD47^−^ killer NPs (CD47-Fc absent) and Blank NPs were prepared and injected (1 mg NPs/mouse) through tail vein into the grafted bm1 recipient mice, respectively, on day 9 post-transplantation. For *in vivo* imaging, the mice were anesthetized by isoflurane inhalation and imaged by using the Maestro system (Cri Inc., Woburn, MA) at 30 min, 2 h, 4 h, 6 h, 12 h, 18 h, 24 h and 30 h after injection. The excitation and emission wavelengths were 635 nm and 665–695 nm, respectively, with 1 min exposure time. At 2 h after injection of ICG-encapsulated killer NPs, the organs (spleen, lungs, heart, liver, lymph nodes and kidneys) were surgically dissected for *ex vivo* imaging.

### *In vivo* distribution of killer NPs and interaction with immune cells

PE-labeled killer NPs were fabricated and injected *i.v.* into recipient bm1 mice on day 9 after skin transplantation. Blood, lymph nodes and spleen were collected at 30 min, 90 min and 4 h after injection, respectively, in the dark. Single-cell suspensions were prepared and then acquired on a FACS Calibur flow cytometer (BD Biosciences).

PE-labeled CD47^+^ and CD47^−^ killer NPs were fabricated as described above and injected into bm1 mice through the tail vein, respectively, on day 9 after skin transplantation. Four hours later, spleens were collected in dark from each group and embedded in freezing medium (O.C.T, Sakura Finetek Inc). Sections with a thickness of 9–10 μm were prepared, de-paraffinized and rehydrated with isopropanol and acetone (1:1 ratio), and then blocked with 10% mouse serum in PBS for overnight at 4 °C. After washing twice with sterile PBS, the sections were incubated with FITC-anti-mouse CD19 (MB19-1, Biolegend, San Diego, CA), FITC-anti-mouse CD4 (RM4-5, eBiosciences), FITC-anti-mouse CD8 (H35-17.2, eBiosciences), FITC-anti-mouse DC11c (N418, eBiosciences) or FITC-anti-mouse F4/80 (m2F8, eBiosciences) at RT for 1 h. After washing with PBS, the sections were further stained with DAPI (Sigma-Aldrich) for 5 min and finally imaged by using confocal laser scanning microscopy (Olympus) after three times washing.

### Enumeration of various immune cells in peripheral blood and spleen

Frequencies of B cells, NK cells, CD3^+^ T cells, CD4^+^ T cells and CD8^+^ T cells were enumerated in spleen cells from the recipient bm1 mice treated by killer NPs, blank NPs or PBS on day 15 after skin graft transplantation. Splenocytes (2 × 10^5^) were stained with PE-anti-mouse CD4 (GK1.5, eBiosciences), APC-anti-mouse CD3e (145-2C11, eBiosciences), FITC-anti-mouse CD19 (MB19-1, Biolegend), FITC-anti-mouse NK1.1 (PK136, Biolegend) and FITC-anti-mouse CD8a (53-6.7, eBiosciences), respectively, for 30 min at 4 °C. The cells were then washed with PBS and analyzed by using flow cytometry and flowJo software. In parallel, peripheral blood was collected from orbital venous of recipient bm1 mice on days 15, 30 and 45 post-transplantation. Routine blood tests were performed by automated hematology analyzer (Sysmex XE-2100, Kobe, Japan).

### Cytotoxicity assay of NK cells

Splenocytes were prepared from recipient bm1 mice on day 15 post-transplantation. A total of 1 × 10^7^ cells were labeled with CFSE as described above and then used as effector cells to co-culture with target cells (Yac-1 cells, 2 × 10^5^ cells/well) at indicated ratios of effector to target in round-bottom 96-well plates in complete RPMI 1640 medium at 37 °C, 5% CO_2_ and humidified conditions for 5 h. Cells were harvested and analyzed by flow cytometry after staining with 7-amino-actinomycin D (7-AAD, eBiosciences). NK activity was calculated as the percentage of 7-AAD-positive cells within a CFSE-negative cell population.

### Tumor cells challenge

The recipient bm1 mice were subcutaneously injected with B16F10 melanoma cells (1 × 10^6^ cells/mouse) in the right groin on day 3 after transplantation and then injected through tail vein with killer NPs, blank NPs or PBS, on days 9, 11 and 13 post-transplantation (1 mg NPs/mouse/time point). Tumor volume was measured daily using a caliper as described by Cao (Cao et al., 2013). Briefly, the tumor in each mouse was measured when it becomes detectable after one week of challenge. Tumors were measured with vernier calipers every two days. The following formula was used to calculate the tumor volume: (the shortest diameter)^2^ × (the longest diameter) × 0.5. The mice were sacrificed, and all visible tumors were excised on day 18.

### Evaluation of organ toxicity

Peripheral blood was collected from the orbital vein of recipient bm1 mice on days 15, 30 and 45 after transplantation. The routine biochemical parameters evaluating hepatic and renal function were detected by automated biochemistry analyzer (Dimension Vista 1500, Siemens Healthcare Diagnostics Inc, Newark, DE). At the same time points, lungs, liver, kidneys, heart and spleen were also harvested from recipient bm1 mice, fixed in 4% formaldehyde and embedded in paraffin. Tissue sections with a thickness of 5–6 μm were prepared and followed by routine H&E staining.

### Statistical analyses

Statistical analyses were performed using the GraphPad Prism 6.0 (GraphPad, La Jolla, CA). To determine the graft survival curve, a Kaplan–Meier graph was constructed, and a log-rank comparison of the groups was used to calculate the *p* values. Wilcoxon signed rank test was used to analyze the tumor sizes. For other experiments, a two-tailed unpaired Student’s *t-*test was used to determine significant differences across groups. All data are presented as the mean ± standard deviation (SD). *p* values < .05 were considered significant.

## Results

### Characterization of PLGA-NPs and phenotypic analyses of killer NPs

Two sizes of PLGA-NPs were prepared here with a spherical shape and a smooth surface morphology (Figure S1A, B). Most of the NPs were 202.4 nm and 78.8 nm in diameter, respectively (Figure S1C, D). The mean zeta potential was −3.09 ± 6.08 mV for 202.4-nm NPs and −8.63 ± 4.59 mV for 78.8-nm NPs (Figure S1E, F).

The maximum amount of protein loaded onto 1 mg of 202.4-nm and 78.8-nm PEI-coated PLGA-NPs was 75.31 µg and 77.51 µg, respectively (Figure S1G, H) as detected by BSA coating assay. The micrographs of confocal microscopy showed that most of the killer NPs generated here co-displayed H-2K^b^-Ig, anti-Fas, PD-L1-Fc and CD47-Fc (Figure S2). TGF-β molecules immobilized onto killer NPs were not detected due to the unavailable of fluorescence-labeled anti-TGF-β antibody.

### Killer NPs treatment markedly prolongs alloskin graft survival and reduces local allo-rejections

**Figure 1.**
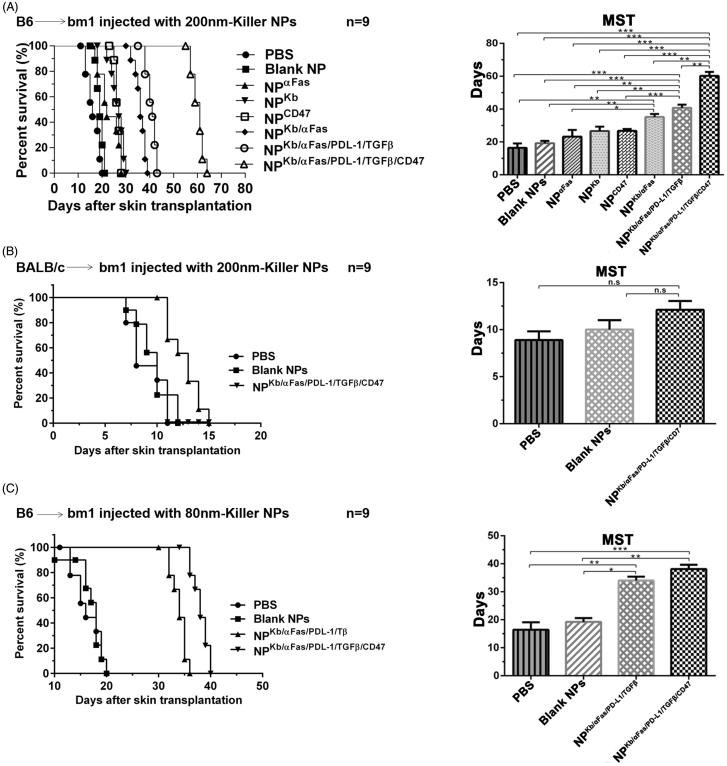
Killer NPs treatment markedly prolongs alloskin graft survival. (A) Kaplan–Meier survival plots for alloskin grafts in the bm1 mice grafted with ear skin of B6 mice. Recipient mice were randomized into 8 groups and injected via tail vein with 200-nm killer NPs or control NPs on days 9, 11 and 13 after skin transplantation. (B) Kaplan-Meier survival plots for alloskin grafts in the bm1 mice grafted with ear skin of BALB/c mice, a third-party alloskin transplant model followed by *i.v.* injection with 200-nm Killer NPs, Blank NPs, or PBS on days 5, 7 and 9 post-transplantation. (C) Kaplan-Meier survival plots for alloskin grafts in the bm1 mice grafted with ear skin of B6 mice. Recipient mice were injected via tail vein with 80-nm killer NPs, NP^Kb/aFas/PD-L1/TGFβ^, Blank NPs or PBS on days 9, 11 and 13 after transplantation. *p < .05, **p < .01, ***p < .001.

As shown in Figure 1(A), the three infusions of H-2K^b^ alloantigen-presenting killer NPs with a diameter of 202.4 nm (termed 200-nm killer NPs) prolonged allograft survival for 45 days with a median survival time (MST) of 61 days as compared with the PBS group. In parallel, the MST of PBS, Blank NPs, NP^aFas^, NP^Kb^, NP^CD47^, NP^Kb/aFas^ and NP^Kb/aFas/PD-L1/TGFβ^ treatment group was 16, 19, 22, 27, 27, 36 and 41 days, respectively. Notably, the CD47^−^ killer NPs (NP^Kb/aFas/PD-L1/TGFβ^) only prolonged the allograft survival for 25 days, nearly half shorter than the CD47^+^ killer NPs (NP^Kb/aFas/PD-L1/TGFβ/CD47^). NP^Kb/aFas/PD-L1/TGFβ^ led to the allograft survival 5 days longer than NP^Kb/aFas^, 14 days longer than NP^Kb^; NP^Kb/aFas^ led to the alloskin graft survival 14 days longer than NP^aFas^. More importantly, the injection of H-2K^b^ alloantigen-presenting killer NPs did not show a remarkable prolongation of alloskin survival as compared to the control groups in the third-party model (Figure 1(B)).

In the case of 78.8-nm killer NPs (termed 80-nm killer NPs), three injections prolonged alloskin survival for 22 days with an MST of 38 days. Meanwhile, the MST of PBS, Blank NPs and NP^Kb/aFas/PD-L1/TGFβ^ group was 16, 18 and 34 days, respectively (Figure 1(C)). The treatment with 200-nm killer NPs was found much more effective than the 80-nm killer NPs in the alloskin transplant model, thus all further experiments were performed by using the 200-nm killer NPs.

**Figure 2.**
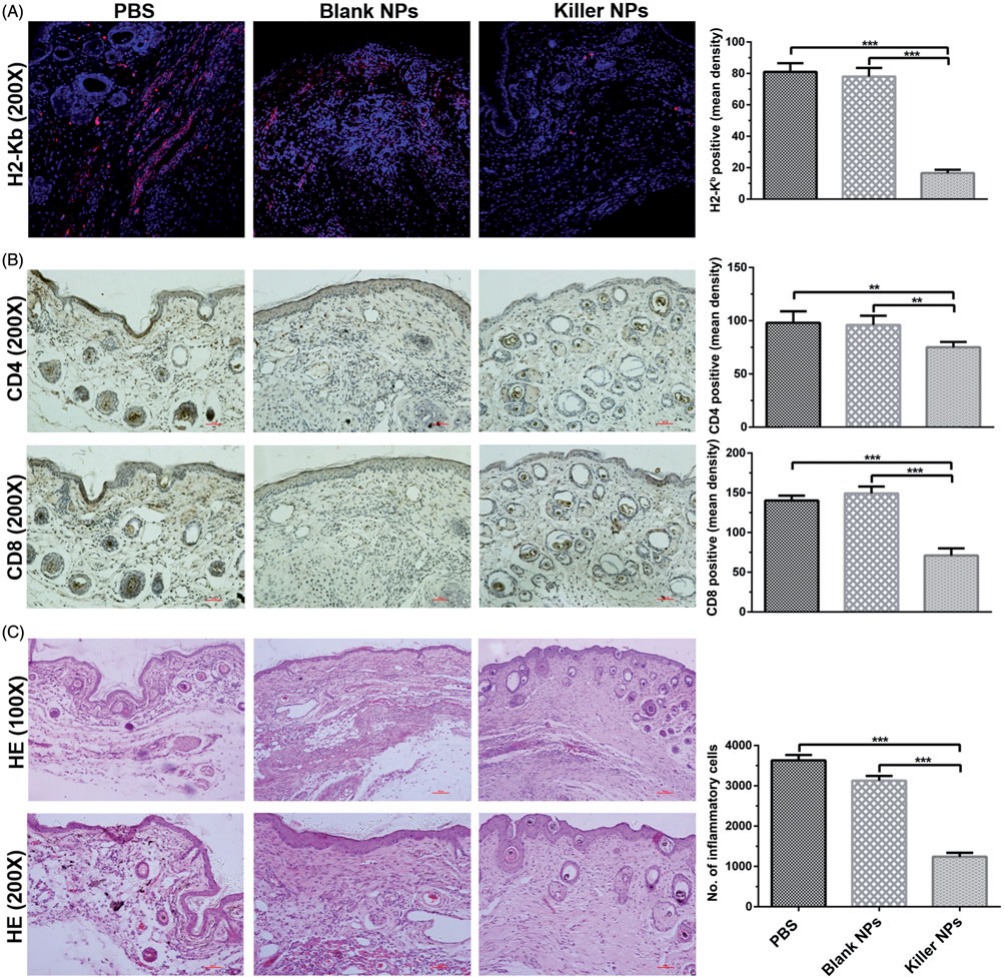
Killer NPs treatment markedly reduces the local allo-rejections of alloskin graft. (A) *In-situ* H-2K^b^-Ig dimer fluorescence staining. (B) IHC staining. (C) H&E staining. Representative images from 3 to 5 individual mice were selected. **p < .01, ***p <  .001.

At 2 days after the final injection of 200-nm killer NPs, the *in situ* H-2K^b^-Ig dimer staining showed around 79.8% reduction of H-2K^b^ alloantigen-specific T cells in the alloskin sections from Killer NPs group, as compared with the Blank NPs and PBS groups (Figure 2(A)). Likewise, the local infiltration of CD8^+^ T cells and CD4^+^ T cells was diminished by 48.9% and 36.4%, respectively (Figure 2(B)). Consistently, only weak inflammatory infiltration was observed in the alloskin sections from Killer NPs group while a strong local inflammation appeared in the control groups as revealed by H&E staining (Figure 2(C)).

### Killer NPs treatment selectively depletes H-2K^b^ alloantigen-reactive CD8^+^ T cells

**Figure 3.**
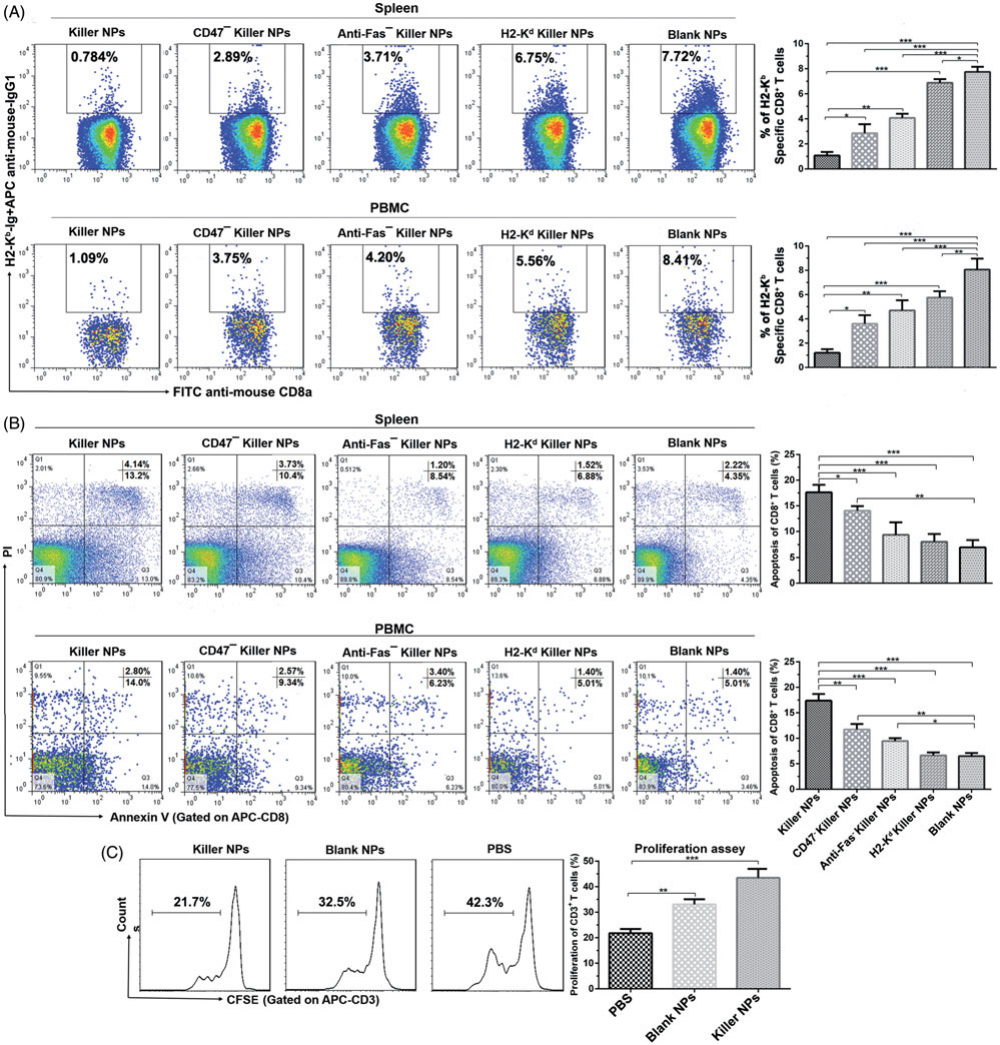
Killer NPs treatment depletes H-2K^b^ alloantigen-reactive CD8^+^ T cell, enhances the apoptosis of CD8^+^ T cells and inhibits the donor-specific alloreactivity of recipient T cells. (A) Frequencies of H-2K^b^ alloantigen-reactive CD8^+^ T cell in the spleens and PMBCs. The percentage of H-2K^b^-Ig dimer^+^/CD8^+^ cells was calculated in the CD8^+^/CD3^+^ cell population. (B) Apoptosis of CD8^+^ T cells in spleen and PBMCs. (C) Anti-donor MLR assays. Splenocytes from recipient bm1 mice were labeled with CSFE first and co-cultured with the Mitomycin C-treated splenocytes from donor B6 mice for 7 days. The proliferation of recipient’s CD3^+^ T cells was determined according to cell division. Data were presented as mean ± SD. *n* = 3 or 4 mice in each group. *p < .05. **p < .01, ***p < .001.

To elucidate the mechanism by which killer NPs prolonged the alloskin survival, the frequencies of H-2K^b^ alloantigen-reactive CD8^+^ T cells in spleen and peripheral blood of recipient bm1 mice was detected by H-2K^b^-Ig dimer staining and flow cytometry. At 2 days after the final injection of 200-nm killer NPs, the proportion of H-2K^b^ alloantigen-reactive CD8^+^ T cells in CD8^+^ T cell population of spleen was 0.748 ± 0.36%, 2.89 ± 0.43%, 3.71 ± 0.63%, 6.75 ± 0.53% and 7.72 ± 0.81% in Killer NPs, CD47^−^ killer NPs, Anti-Fas^−^ killer NPs, H2-K^d^ killer NPs and Blank NPs groups, respectively (Figure 3(A)). As compared with Blank NPs group, Killer NPs (NP^Kb/aFas/PD-L1/TGFβ/CD47^) treatment led to 90.3% off the frequency of H-2K^b^-alloreactive CD8^+^ T cells in spleen, while the CD47^−^ killer NPs (NP^Kb/aFas/PD-L1/TGFβ^) and Anti-Fas^−^ killer NPs (NP^Kb/PD-L1/TGFβ/CD47^) resulted in a 62.6% and 51.9% reduction, respectively. More importantly, the H-2K^d^ alloantigen-presenting killer NPs (H-2K^d^ killer NPs), which displays a noncognate alloantigen in this transplantation model, only caused a 12.6% reduction of the H-2K^b^-alloreactive CD8^+^ T cells when compared to Blank NPs group. Consistently, the killer NPs treatment also led to 87.0% off the frequency of H-2K^b^-alloreactive CD8^+^ T cells in the peripheral blood (PBMCs) of recipients at the same time point (Figure 3(A)). These data documented the selective reduction of H-2K^b^ alloantigen-reactive CD8^+^ T cells by the killer NPs treatment in spleen and peripheral blood, which consequently cause the diminished local infiltration of these cells in the alloskin graft.

### Killer NPs treatment facilitates the apoptosis of CD8^+^ T cells, inhibits the activation and alloreactivity of T cells and induces regulatory T cells

The mechanisms by which killer NPs reduced the H2-K^b^ alloantigen-reactive CD8^+^ T cells were further investigated. Two days after the final injection of killer NPs, spleen, peripheral blood and lymph nodes were collected from each treatment group. In spleen, the mean percentage of apoptotic CD8^+^ T cells in the population of CD8^+^ T cells was 17.34 ± 0.8%, 14.03 ± 0.66%, 9.74 ± 1.39%, 8.40 ± 0.39% and 6.57 ± 0.43% in Killer NPs, CD47^−^ killer NPs, Anti-Fas^−^ killer NPs, H2-K^d^ killer NPs and Blank NPs groups, respectively (Figure 3(B)). When compared to Blank NPs group, the killer NPs treatment caused nearly threefold increased apoptosis of CD8^+^ T cells. Meanwhile, the apoptosis of CD8^+^ T cells increased by 113.5% in CD47^−^ killer NPs group and 48.2% in Anti-Fas^−^ killer NPs group. But the no-cognate H-2K^d^ killer NPs only caused little higher apoptosis of CD8^+^ T cells than the blank NPs, without significant difference. In peripheral blood, about two-fold increase of apoptotic CD8^+^ T cells was observed in Killer NPs group, while a 96.2% increase in CD47^−^ killer NPs group and a 58.6% increase in Anti-Fas^−^ killer NPs groups (Figure 3(B)).

**Figure 4.**
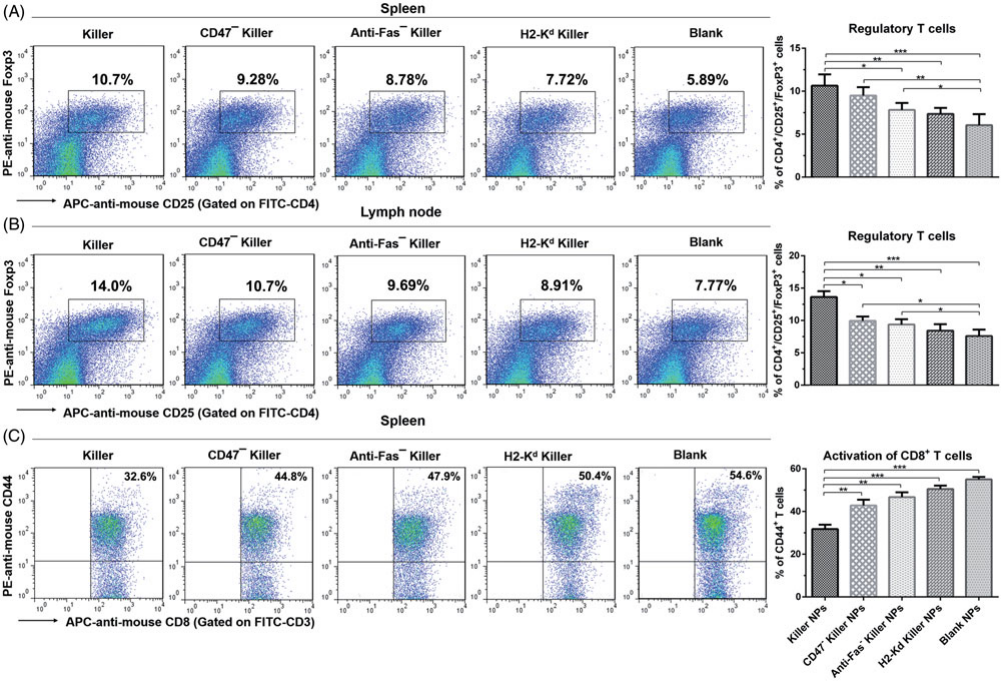
Killer NPs treatment induces the regulatory T cells and inhibits the activation of CD8^+^ T cells. (A, B) Tregs in spleen and lymph nodes. (C) Activation of CD8^+^ T cells in spleen. Data were presented as mean ± SD. *n* = 3 or 4 mice in each group. *p < .05. **p < .01, ***p < .001.

The proliferation of recipient T cells in response to donor splenocytes was markedly inhibited by about 50% in Killer NPs group relative to the recipient T cells from PBS group (Figure 3(C)), as presented by the anti-donor MLR assay. As shown in Figure 4(A,B), a significantly higher proportion of CD4^+^/CD25^+^/Foxp3^+^ regulatory T cells in CD4^+^ T cell population was found in spleen and lymph nodes in the killer NPs group, with an increase of 81.7% in spleen and 80.1% in lymph nodes relative to Blank NPs group. In addition, Killer NPs treatment significantly inhibited the activation of CD8^+^ T cells with a decrease of 67.5% relative to Blank NPs group (Figure 4(C)).

### *In vivo* tracking and tissue distribution of killer NPs

**Figure 5.**
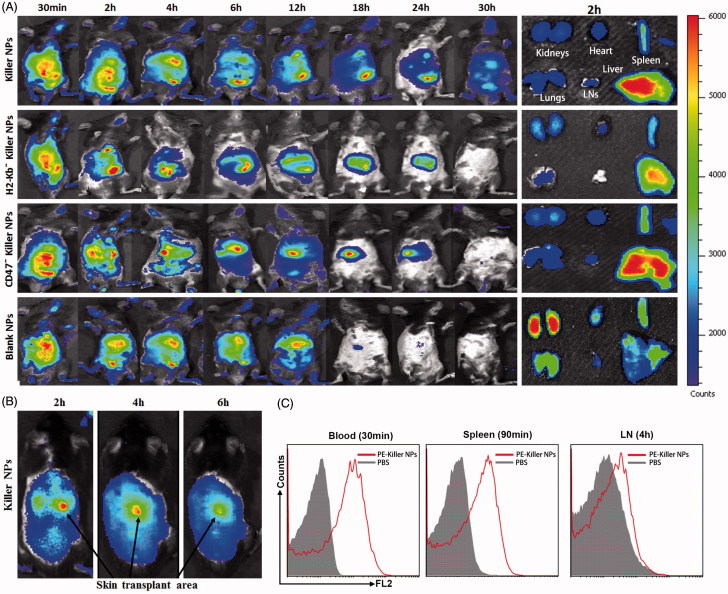
*In vivo* tracking and tissue distribution of killer NPs. ICG-encapsulated killer NPs, H2-K^b−^ killer NPs, CD47^−^ killer NPs or blank NPs were *i.v.* administered to recipient bm1 mice on day 9 after skin transplantation. (A) Whole-body *in vivo* imaging (left panel) and e*x vivo* imaging for the excised organs at 2 h after injection (right panel). (B) The localization of killer NPs in alloskin grafts by whole-body fluorescence imaging. (C) PE-labeled killer NPs presented in peripheral blood, spleen and lymph nodes at indicated time points as detected by flow cytometry.

Grafted bm1 mice were injected via the tail vein with ICG-encapsulated killer NPs, H-2K^b-^ killer NPs (NP^aFas/PD-L1/TGFβ/CD47^), CD47^−^ killer NPs (NP^Kb/aFas/PD-L1/TGFβ^) and blank NPs, respectively, and followed by *in vivo* and *ex vivo* near-infrared imaging. Whole-body imaging showed the rapid and selective accumulation of ICG-encapsulated killer NPs in spleen, liver, lungs, kidney, heart and lymph nodes. The strongest fluorescent intensity was observed in mice from 30 min to 4 h after *i.v*. administration, with a retention time up to 30 h. As controls, H-2K^b−^ killer NPs (non-targeting killer NPs), CD47^−^ killer NPs and Blank NPs showed the *in vivo* trafficking a little different from the killer NPs, and a shorter retention time (24 h, 24 h and 18 h, respectively) (Figure 5(A)). At 2-h time point after injection, the *ex vivo* imaging of excised organs demonstrated that the killer NPs only appeared in liver and spleen, H-2K^b−^ killer NPs and CD47^−^ killer NPs mainly accumulated in liver and spleen while less in kidneys, but the blank NPs widely presented in kidneys, lungs, spleen, liver and heart (Figure 5(A), right panel). These differences may imply that H-2K^b^ and CD47 molecules enable the killer NPs to target H-2K^b^ alloantigen-specific T cells and resist phagocytosis, thus make the *in vivo* trafficking and tissue distribution distinct to the H-2K^b−^ killer NPs and CD47^−^ killer NPs. Notably, the killer NPs were observed at the location of alloskin graft at 2, 4 and 6 h after injection (Figure 5(B)), suggesting that the 200-nm killer NPs could go into the alloskin graft through vascular circulation at least during the early 6 hr. Furthermore, the detection by flow cytometry also confirmed the presence of PE-labeled killer NPs in peripheral blood at 30 min, spleen at 90 min and lymph nodes at 4 hr (Figure 5(C)).

### Colocalization of killer NPs with CD8^+^ T cells *in vivo*

**Figure 6.**
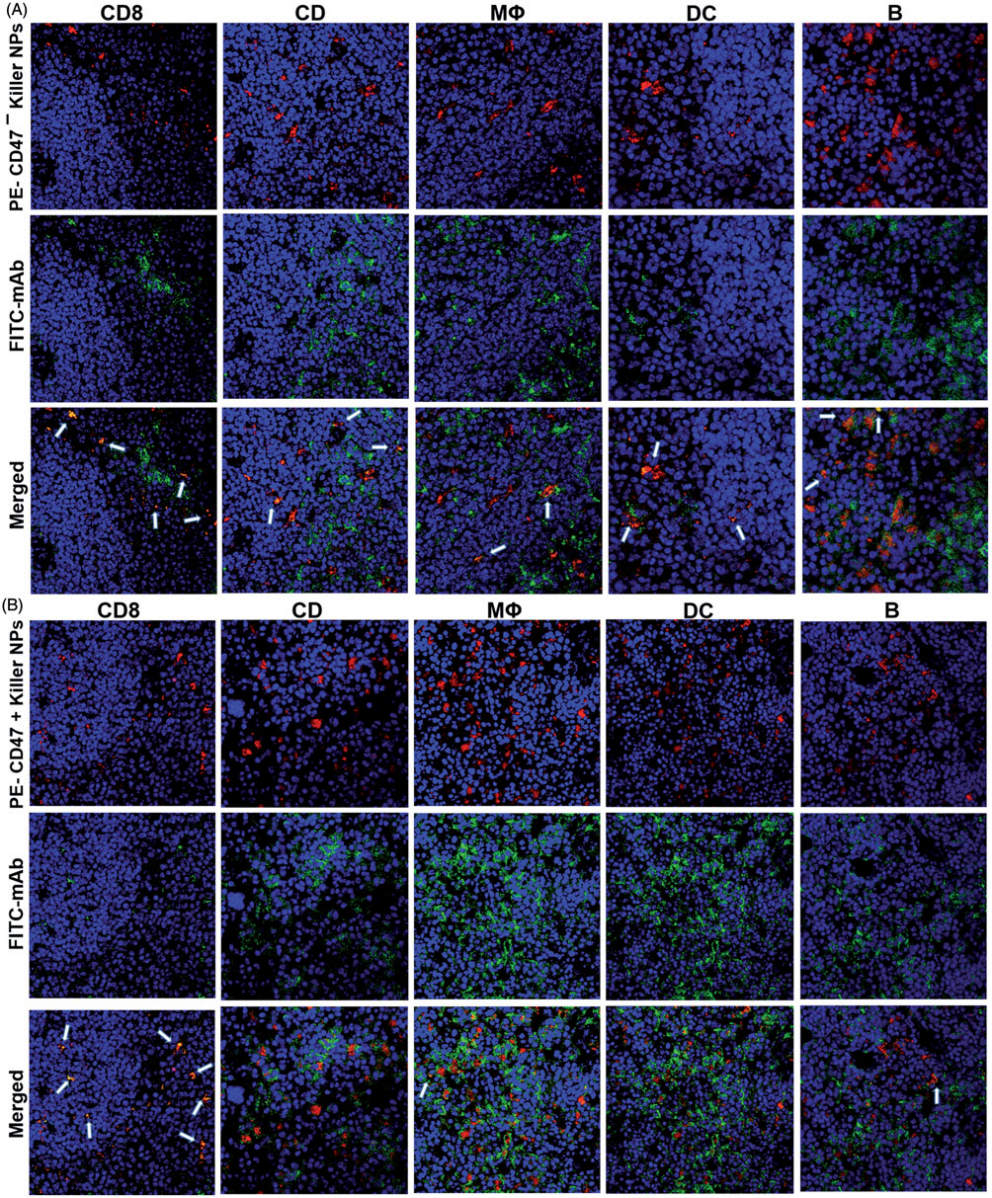
Confocal fluorescence imaging for the co-localizations of killer NPs with immune cells in spleen sections. On day 9 after skin transplantation, PE-coupled CD47^+^ and CD47^−^ killer NPs were *i.v.* injected, respectively, into recipient bm1 mice, and spleens were harvested in dark after 3 h. The frozen sections were stained with FITC-labeled mAbs specific for CD8^+^ T cells, CD4^+^ T cells, macrophages, dendritic cells, or B cells. Confocal photomicrographs of the immune cells with PE-CD47^−^ killer NPs (A) and PE-CD47^+^ killer NPs (B) were presented, respectively, at 400 × magnification.

To investigate whether the killer NPs can contact locally with alloreactive T cells and be internalized by phagocytes in secondary lymphoid organs, PE-labeled killer NPs and CD47^−^ killer NPs were injected *i.v.*, respectively, into the grafted bm1 mice on day 9 after alloskin transplantation. The frozen sections of spleen isolated at 4 h after injection were processed for immunofluorescence staining. The confocal fluorescence images demonstrated that PE-CD47^−^ killer NPs mainly distributed in the red pulp and marginal zone and presented many colocalizations with CD8^+^ T cells, CD4^+^ T cells, macrophage and dendritic cells as well as fewer contacts with B cells (Figure 6(A)). However, more PE-CD47^+^ killer NPs were observed in the red pulp and marginal zone and displayed much more co-localizations with CD8^+^ T cells, but much fewer contacts with other cells than PE-CD47^−^ killer NPs (Figure 6(B)). These results indicate the direct contacts of killer NPs with CD8^+^ T cells and very less engulfment by macrophage and dendritic cells *in vivo*.

### Killer NPs treatment does not produce obvious bystander killing to immune cells

As compared with the PBS and blank NPs groups, the killer NPs treatment did not significantly decrease the frequencies of CD3^+^ T cells, CD4^+^ T cells, B cells and NK cells, but led to a visible reduction of CD8^+^ T cells in the spleen cell suspensions on day 15 (Figure S3). Furthermore, blood routine tests were performed on days 15, 30 and 45 (2 days, 17 days and 32 days after the final injection) in another independent experiment. No obvious bystander killing to the eight cell populations in peripheral blood was found at each time point after the killer NPs treatment (Figure S4A).

### Killer NPs treatment does not impair overall host immune function and presents no apparent organ toxicity

Antitumor effect and alloreactivity are usually used as surrogate markers for the retention of overall host immune function (Brandhorst et al., 2013). On day 15, the recipient splenocytes were collected from each group and followed by a third-party MLR assay. A comparable proliferation level of recipient T cells was observed between the killer NPs group and PBS group (Figure S5A, B). Similarly, the cytolysis level of recipient NK cells against Yac-1 lymphoma cells was also comparable across groups (Figure S5C). After B16F10 melanoma cell challenge, the comparable tumor growth was found in the killer NPs, blank NPs and PBS treatment groups (Figure S6).

On days 15, 30 and 45, the functions of liver and kidney of recipient bm1 mice were not impaired significantly by killer NPs treatment as monitored by routine biochemical tests (Figure S4B, C). Meanwhile, no visible damage was observed in spleen (Figure S7A), kidney (Figure S7B), liver (Figure S7C), heart (Figure S7D) and lungs (Figure S7E) in recipient bm1 mice after killer NPs treatment as analyzed by H and E staining at same time points. Both functional and histopathological analyses suggest that the killer NPs treatment did not cause apparent organ toxicity at the long-time points in the alloskin transplant murine model.

## Discussion

The rapid progress of biomaterials greatly facilitates the researches using biomimetic NPs as modulators in autoimmunity and allograft rejection by coating or encapsulating antigens, toxins and/or cytokines (Tsai et al., 2010; Getts et al., 2012; Bryant et al., 2014; Fisher et al., 2015; Hotaling et al., 2015; Maldonado et al., 2015). But most of the nanoparticle-based therapeutics underlay the antigen presentation by cellular uptake of NPs and the following induction of tolerogenic APCs and regulatory T cells, where after indirectly induce T-cell tolerance. The indirect modulations on antigen-specific T cells often suffer from the unstable induction of tolerogenic APCs *in vivo* due to their diverse types, tissue specificities and surface receptors (Tsai et al., 2010; Balmert & Little, 2012; Bryant et al., 2014; Fisher et al., 2015; Hartwell et al., 2015). Unlike these previous works, this study focus on the antigen-specific contacts of NPs with T cells via surface presentation of ligands and the combined use of multiple regulatory molecules. Here, H-2K^b^-Ig dimer was presented as target antigen which could bind directly with the TCR of H-2K^b^ alloantigen-specific CD8^+^ T cells, thus enacting the antigen-specific and direct depletion and modulation of alloreactive T cells. Anti-Fas, PD-L1 and TGF-β were confirmed to induce apoptosis, inhibit activation and proliferation of T cells or induce regulatory T cells through different signal pathways. To minimize the uptake of NPs by phagocytes, the 200-nm killer NP was coated with the CD47-Fc, which can serve as “don't eat me” signal (Oldenborg et al., 2000; Poon et al., 2014) and was used to construct the “stealth particles” in NP-based drug delivery system to increase the circulation time of NP delivery vehicles (Massarelli et al., 2014). As shown by our data, the CD47^+^ killer NPs prolonged the allograft survival for 45 days, almost twofold duration relative to the CD47^−^ killer NPs (25 days), and presented the much stronger effects on depleting H-2K^b^-alloreactive CD8^+^ T cells, inducing apoptosis and Tregs, and inhibiting the activation and allo-proliferation of T cells, in comparison with the CD47^−^ killer NPs. Moreover, the CD47^+^ killer NPs displayed a much longer retention time *in vivo*, and much more contacts with CD8^+^ T cells and fewer colocalizations with macrophages, dendritic cells and B cells than the CD47^−^ killer NPs. As expected, CD47 molecule remarkably enhanced the therapeutic effects of the killer NPs, thus tailored that most of the killer NPs could directly present the intact MHC class I alloantigen and multiple regulatory molecules to the alloreactive CD8^+^ T cells in the same spatial and temporal context, by minimizing the *in vivo* engulfment of NPs by phagocytes. Of course, we cannot eliminate the possibility of pinocytosis and phagocytosis occurring *in vivo*. Therefore, it is reasonable to conclude that the killer NPs modulate the alloreactive T cells *in vivo* mainly in a direct contact way, but an indirect pathway mediated by regulatory T cells and tolerogenic APCs may also involve in this immunotherapy.

Biomimetic MPs also attract the attention for immunotherapy. In our previous works, the killer PLGA-MPs were generated by covalently cocoupling H-2K^b^-Ig dimers and anti-Fas onto 4.5-μm PLGA-MPs. Intravenous injections could markedly prolong the alloskin graft survival by selectively depleting alloreactive CD8^+^ T cells in a single MHC-mismatched murine model (Wang et al., 2017). In the present study, two points are different from our previous works. Firstly, the 200-nm PLGA-NPs rather than the cell-sized particles were used as an acellular scaffold. When compared with the MP-based KaAPCs, the present 200-nm killer NPs displayed a much higher accumulation in lymph nodes at 90 min and spleen at 4 h as detected by flow cytometry. More importantly, the killer NPs were observed in the local alloskin graft during the early 6 h after *i.v.* injection. Reversely, the 4.5-μm killer MPs cannot go to the alloskin location (Wang et al., 2017). Secondly, in addition to H-2K^b^-Ig dimer and anti-Fas mAb, the PD-L1-Fc, TGF-β and CD47-Fc were also cocoupled onto the surface of killer NPs to obtain a powerful immune inhibition through multiple signal pathways and minimized uptake of NPs *in vivo*. However, under the current regimens, the killer NPs carrying multiple regulatory molecules presented the therapeutic outcomes similar to the killer MPs which only carrying anti-Fas. The nanoscale and microscale killer particles prolonged the alloskin survival for 45 and 43 days, respectively. This no difference may owe to the size effects of biomimetic particles. As known, the size of micro- and nanoparticles (MNPs) greatly affect the ability of MNPs to pass through biological barriers, be engulfed by phagocytes and interplay with target cells via surface presentation of ligands (Balmert & Little, 2012). The closer the particle is to cellular size, the more potent the effect on the target cell (Mescher, 1992; Meyer et al., 2015a). Cell-sized MPs present a reduced risk of engulfment by phagocytes relative to nanoparticles (Champion et al., 2008; Balmert & Little, 2012). Here, the killer NPs may be more easily to be internalized than killer MPs even if the presence of CD47 molecules. However, the most important advantage of the killer NPs is the much less risk of embolism than the cell-sized killer MPs after *i.v.* injection, thus will be more applicable to the translational studies than the microscale counterpart. Of note is that even smaller NPs (< 100 nm) will lose the therapeutic potential as shown by the 80-nm killer NPs in the current study because they can pass through endothelial barriers (Perrault et al., 2009) and be internalized by pinocytosis even if decorated with CD47 molecules.

Finally, several technical notes are worthy of mentioning. In this study, a single-MHC mismatched alloskin transplant model was employed. It is a weak immunogenic murine model of allograft, but the single mutation (K^b^) between C57BL/6 J and bm1 mice is sufficient to cause the acute alloskin rejection with an MST of 19.5 ± 3 days (Isakov & Segal, 1983; Wang et al., 2010). More importantly, it can maximally reveal the therapeutic outcome of killer NPs without the interference from the alloantigen responses against other mismatched MHC and minor histocompatibility loci. Of note is that several control NPs were performed along with the killer NPs in this study and defined the contributions of each kind of molecules carried by the killer NPs, suggested the antigen-specific manner and regulatory molecule-dependent fashion of the killer NPs treatment. Although our results showed promising prospects of the killer NPs in immunotherapy, many aspects still need to be further investigated for the approaches from bench to bedside. All the MHC class I and class II alloantigens mismatched between recipient and donor should be composed onto the killer NPs in translational studies; the density and ratios of MHC alloantigens and multiple regulatory molecules immobilized onto NPs should be further titrated to achieve maximal antigen-specific modulation on T cells with minimal bystander killing.

In conclusion, our preclinical data demonstrate that the 200-nm PLGA-NPs carrying MHC alloantigen, multiple regulatory molecules and “self-marker” are capable of prolonging allograft survival by directly and selectively modulating alloreactive T cells. The *in vivo* mechanism of alloinhibition, tissue distribution and toxicity were also initially defined. This strategy may pave a new avenue for the treatment of allograft rejections.

## Supplementary Material

IDRD_Shen_et_al_Supplemental_Content.zip
