# Supplementary material for: On-target and direct modulation of alloreactive T cells by a nanoparticle carrying MHC alloantigen, regulatory molecules and CD47 in a murine model of alloskin transplantation
Source: Drug Deliv. 2018 Mar 6;25(1):703–15. doi: 10.1080/10717544.2018.1447049 (PMC6058602; doi:10.1080/10717544.2018.1447049)
Supplement: IDRD_Shen_et_al_Supplemental_Content.zip [file IDRD_A_1447049_SM2178.zip › Supplementary Figure 1.pdf]

Supplementary Figure 1:

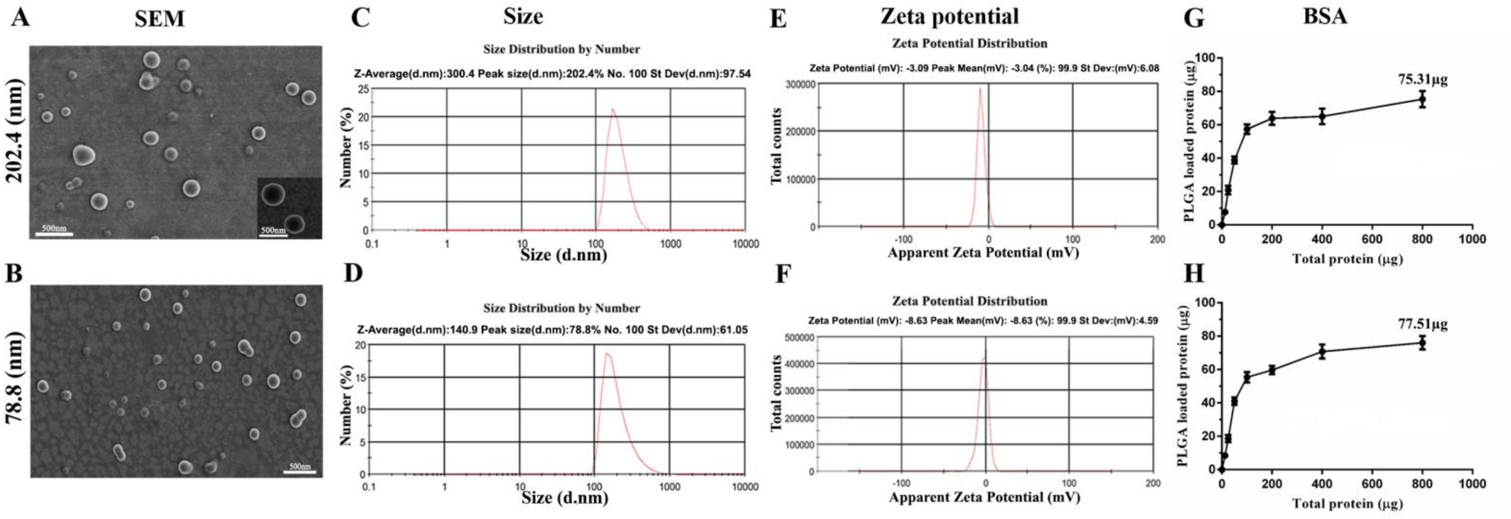

Fig. S1 Preparation and characterization of two-sized PLGA-NPs. (A, B) Representative SEM micrographs. (C, D) Size distribution. (E, F) Zeta potential distribution. (G, H) The protein coupling capability of 202.4-nm and 78.8-nm PLGA-NPs as detected by micro BCA assay.
