# Supplementary material for: On-target and direct modulation of alloreactive T cells by a nanoparticle carrying MHC alloantigen, regulatory molecules and CD47 in a murine model of alloskin transplantation
Source: Drug Deliv. 2018 Mar 6;25(1):703–15. doi: 10.1080/10717544.2018.1447049 (PMC6058602; doi:10.1080/10717544.2018.1447049)
Supplement: IDRD_Shen_et_al_Supplemental_Content.zip [file IDRD_A_1447049_SM2178.zip › Supplementary Figure 2.pdf]

Supplementary Figure 2:

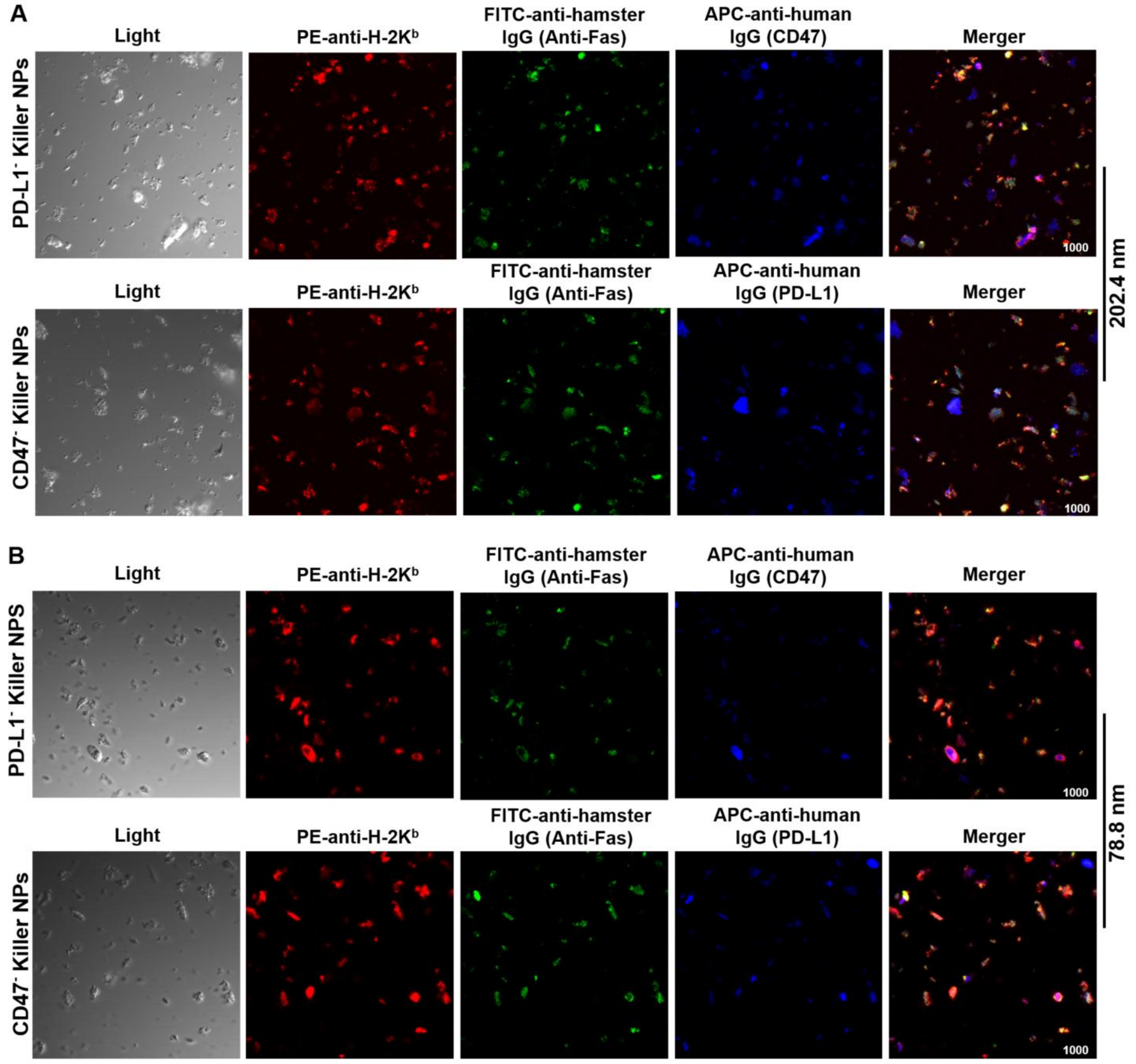

Fig. S2 Phenotypic analyses of killer PLGA-NPs. Since the APC-anti-human IgG mAb can bind to both CD47-Fc and PD-L1-Fc, the CD47$^-$ killer NPs (NP$^{Kb/aFas/PD\text{-}L1/TGF\beta}$) and PD-L1$^-$killer NPs (NP$^{Kb/aFas/TGF\beta/CD47}$) were prepared respectively and stained with PE-anti-H-2K^b, FITC-anti-hamster IgG (binding to anti-Fas) and APC-anti-human-IgG. After washing, the killer NPs were imaged using confocal laser scanning microscope. (A) Phenotype analyses of 202.4-nm killer NPs. (B) Phenotype analyses of 78.8-nm killer NPs. TGF-β molecules immobilized onto killer NPs were not detected due to the unavailable of fluorescence-labeled anti-TGF-β antibody.
