# Supplementary material for: On-target and direct modulation of alloreactive T cells by a nanoparticle carrying MHC alloantigen, regulatory molecules and CD47 in a murine model of alloskin transplantation
Source: Drug Deliv. 2018 Mar 6;25(1):703–15. doi: 10.1080/10717544.2018.1447049 (PMC6058602; doi:10.1080/10717544.2018.1447049)
Supplement: IDRD_Shen_et_al_Supplemental_Content.zip [file IDRD_A_1447049_SM2178.zip › Supplementary Figure 3.pdf]

**Supplementary Figure 3:**

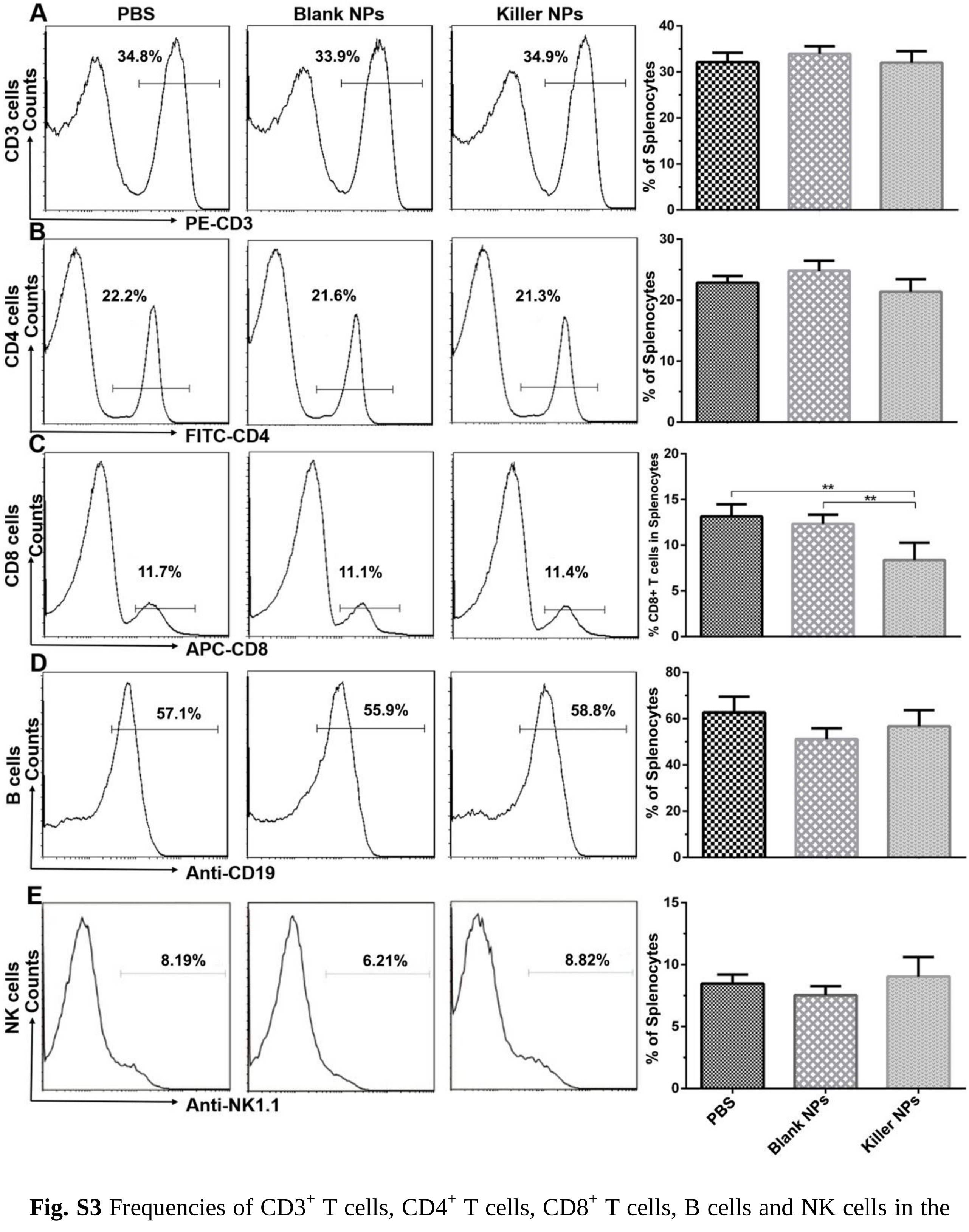


**Fig. S3** Frequencies of $CD3^+$ T cells, $CD4^+$ T cells, $CD8^+$ T cells, B cells and NK cells in the recipient spleen cell suspensions after treatment with killer NPs. Spleens were harvested from the recipient bm1 mice in each group on day 15 after skin transplantation (2 days after the final injection of killer NPs, blank NPs, or PBS). Splenocytes were stained with

APC-anti-mouse CD3e, PE-anti-mouse CD4, FITC-anti-mouse CD8a, FITC-anti-mouse CD19, FITC-anti-mouse NK1.1, and isotype control mAbs, respectively, and followed by flow cytometry. The frequencies and representative histograms of $CD3^+$ T cells (A), $CD4^+$ T cells (B), $CD8^+$ T cells (C), B cells (D) and NK cells (E) in each group were displayed. Data were presented as mean ± SD. n = 3 or 4 mice in each group. ***p*<0.01.
