# Supplementary material for: On-target and direct modulation of alloreactive T cells by a nanoparticle carrying MHC alloantigen, regulatory molecules and CD47 in a murine model of alloskin transplantation
Source: Drug Deliv. 2018 Mar 6;25(1):703–15. doi: 10.1080/10717544.2018.1447049 (PMC6058602; doi:10.1080/10717544.2018.1447049)
Supplement: IDRD_Shen_et_al_Supplemental_Content.zip [file IDRD_A_1447049_SM2178.zip › Supplementary Figure 4.pdf]

Supplementary Figure 4:

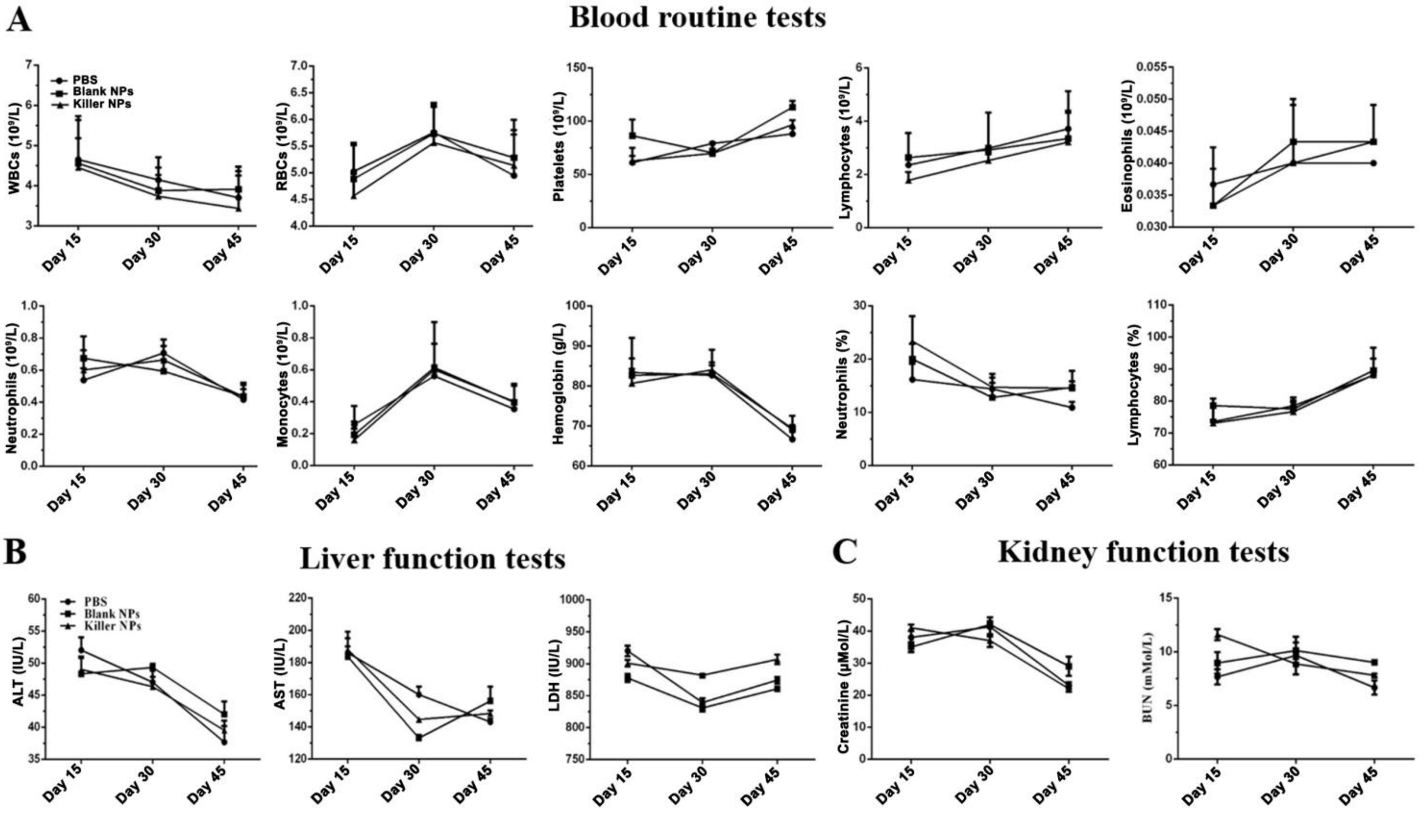

Fig. S4 Blood routine tests and functional evaluation of liver and kidney at different time intervals after treatment with killer NPs. Peripheral blood samples were harvested from the recipient bm1 mice in each group on days 15, 30 and 45 after skin transplantation (2, 17 and 32 days after the final injection of killer NPs, blank NPs, or PBS). For each sample, 200 µl of blood was used for blood routine tests, and rest of the blood (about 600µl) was processed for serum isolation and biochemical tests. (A) Frequencies of white blood cells (WBCs), red blood cells (RBCs), platelets, lymphocytes, eosinophils, neutrophils, monocytes and the level of hemoglobin in peripheral blood at time points in each group. (B, C) Routine biochemical tests for the functional analyses of kidney and liver at time points in each group. ALT: alanine aminotransferase, AST: aspartate aminotransferase, LDH: lactate dehydrogenase, BUN: blood urea nitrogen and creatinine. Data were presented as mean ± SD. n = 3 or 4 mice per group at each time point. No significant difference was found across groups.
