# Supplementary material for: On-target and direct modulation of alloreactive T cells by a nanoparticle carrying MHC alloantigen, regulatory molecules and CD47 in a murine model of alloskin transplantation
Source: Drug Deliv. 2018 Mar 6;25(1):703–15. doi: 10.1080/10717544.2018.1447049 (PMC6058602; doi:10.1080/10717544.2018.1447049)
Supplement: IDRD_Shen_et_al_Supplemental_Content.zip [file IDRD_A_1447049_SM2178.zip › Supplementary Figure 5.pdf]

Supplementary Figure 5:

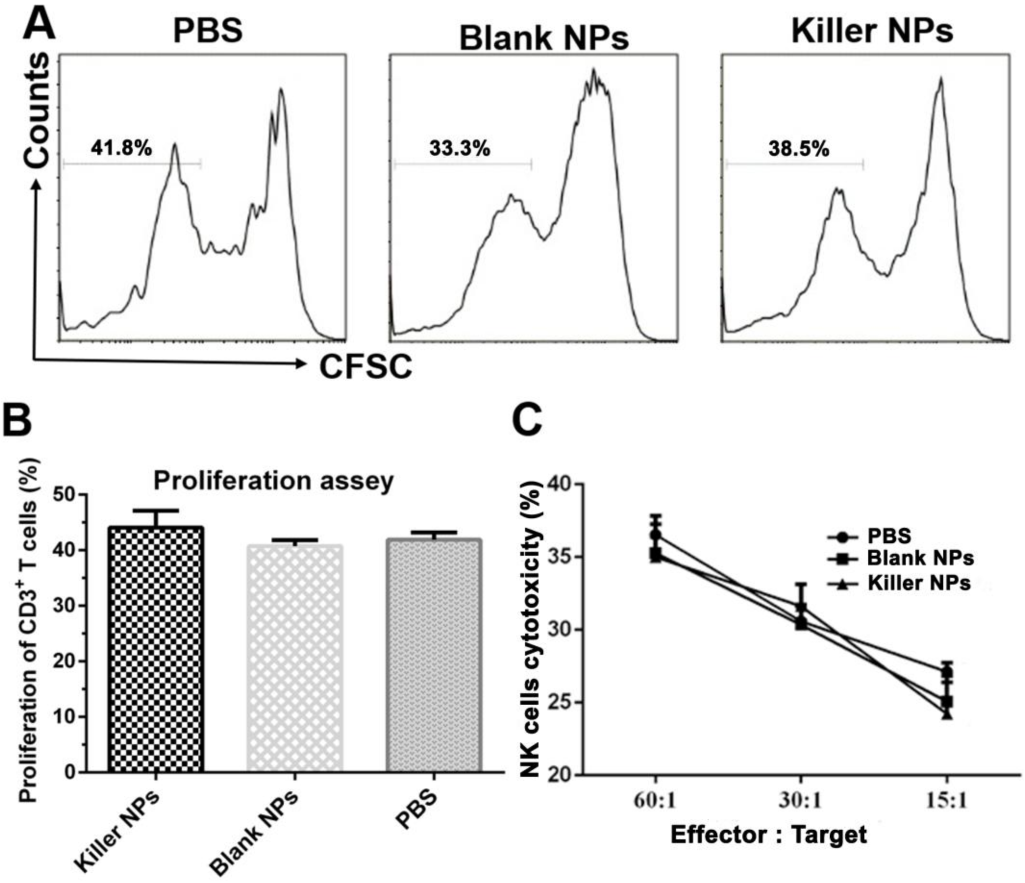

Fig. S5 Third-party-specific alloreactivity of recipient T cells and cytotoxicity of recipient NK cells after treatment with killer NPs. On day 15 after skin transplantation (two days after the final injection of killer NPs, blank NPs or PBS), the spleens from recipients of each group were harvested and processed to single cell suspensions. For a third-party MLR assay, the splenocytes were labeled with CFSE first and then co-incubated with the mitomycin C-treated splenocytes from BALB/c mice for 7 days in 96-well plate. After that, the cells were collected, stained with PE-anti-mouse CD3e and followed by flow cytometry. The percentage of proliferation for recipient $CD3^+$ T cells was determined according to cell division. (A) Representative histograms for the division of recipient T cells in the third-party MLR assay. (B) Proliferation level of recipient T cells in response to the third-party alloantigen in each group. For cytotoxicity assay of NK cells, splenocytes were labeled with CFSE first and then co-incubated with Yac-1 cells at indicated ratios of effector to target cells in 96-well plates for 5 hr. Cells were harvested and analyzed by flow cytometry after staining with 7-AAD. NK activity was calculated as the percentage of 7-AAD-positive cells within a CFSE-negative cell population and presented in (C). Data were presented as mean ± SD. n = 4 mice per group.
