# Supplementary material for: On-target and direct modulation of alloreactive T cells by a nanoparticle carrying MHC alloantigen, regulatory molecules and CD47 in a murine model of alloskin transplantation
Source: Drug Deliv. 2018 Mar 6;25(1):703–15. doi: 10.1080/10717544.2018.1447049 (PMC6058602; doi:10.1080/10717544.2018.1447049)
Supplement: IDRD_Shen_et_al_Supplemental_Content.zip [file IDRD_A_1447049_SM2178.zip › Supplementary Figure 6.pdf]

Supplementary Figure 6:

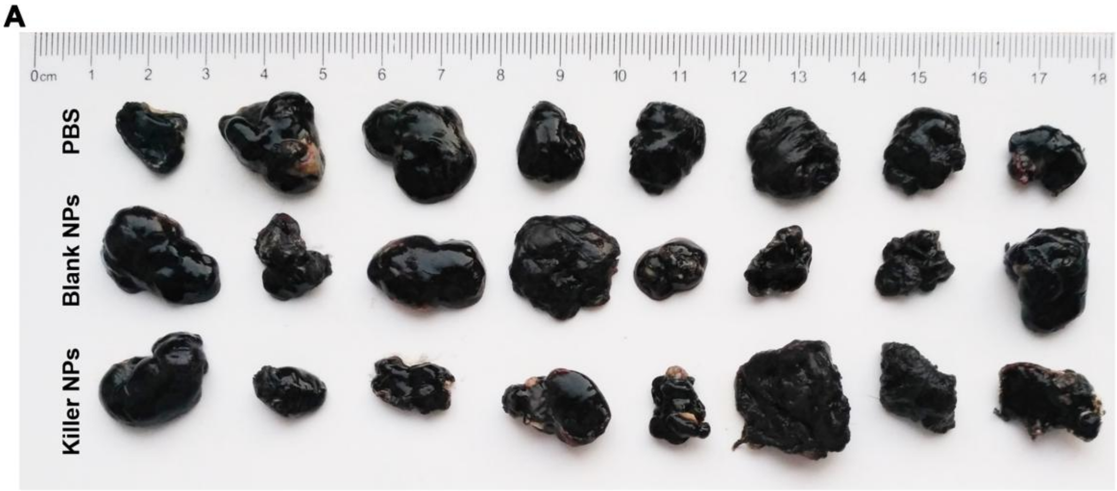

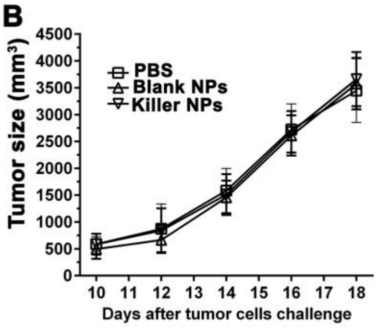

Fig. S6 Antitumor effects of recipient bm1 mice after treatment with killer NPs. On day 3 after skin transplantation, the recipient bm1 mice were subcutaneously injected with melanoma B10F10 mouse cells in the right groin, and then injected through tail vein with killer NPs, blank NPs or PBS, on days 9, 11 and 13 post transplantation. Tumor volume was measured daily using a caliper. Briefly, the tumor in each mouse was measured when it became detectable after one week of challenge. Tumors were measured with venire calipers every two days. The following formula was used to calculate the tumor volume: (the shortest diameter)^2 × (the longest diameter) × 0.5. The mice were sacrificed, and all visible tumors were excised. (A) Excised tumors from each mouse in each group on day 18 after tumor inoculation. (B) Growth curves of tumors in each group during 18 days after tumor inoculation. The differences across groups were analyzed by using Wilcoxon signed rank test. The killer NPs treatment did not obviously impair the overall anti-tumor effects of recipient bm1 mice. Data were presented as mean ± SD. n = 8 mice in each group.
