# Supplementary material for: On-target and direct modulation of alloreactive T cells by a nanoparticle carrying MHC alloantigen, regulatory molecules and CD47 in a murine model of alloskin transplantation
Source: Drug Deliv. 2018 Mar 6;25(1):703–15. doi: 10.1080/10717544.2018.1447049 (PMC6058602; doi:10.1080/10717544.2018.1447049)
Supplement: IDRD_Shen_et_al_Supplemental_Content.zip [file IDRD_A_1447049_SM2178.zip › Supplementary Figure 7.pdf]

Supplementary Figure 7:

Spleen

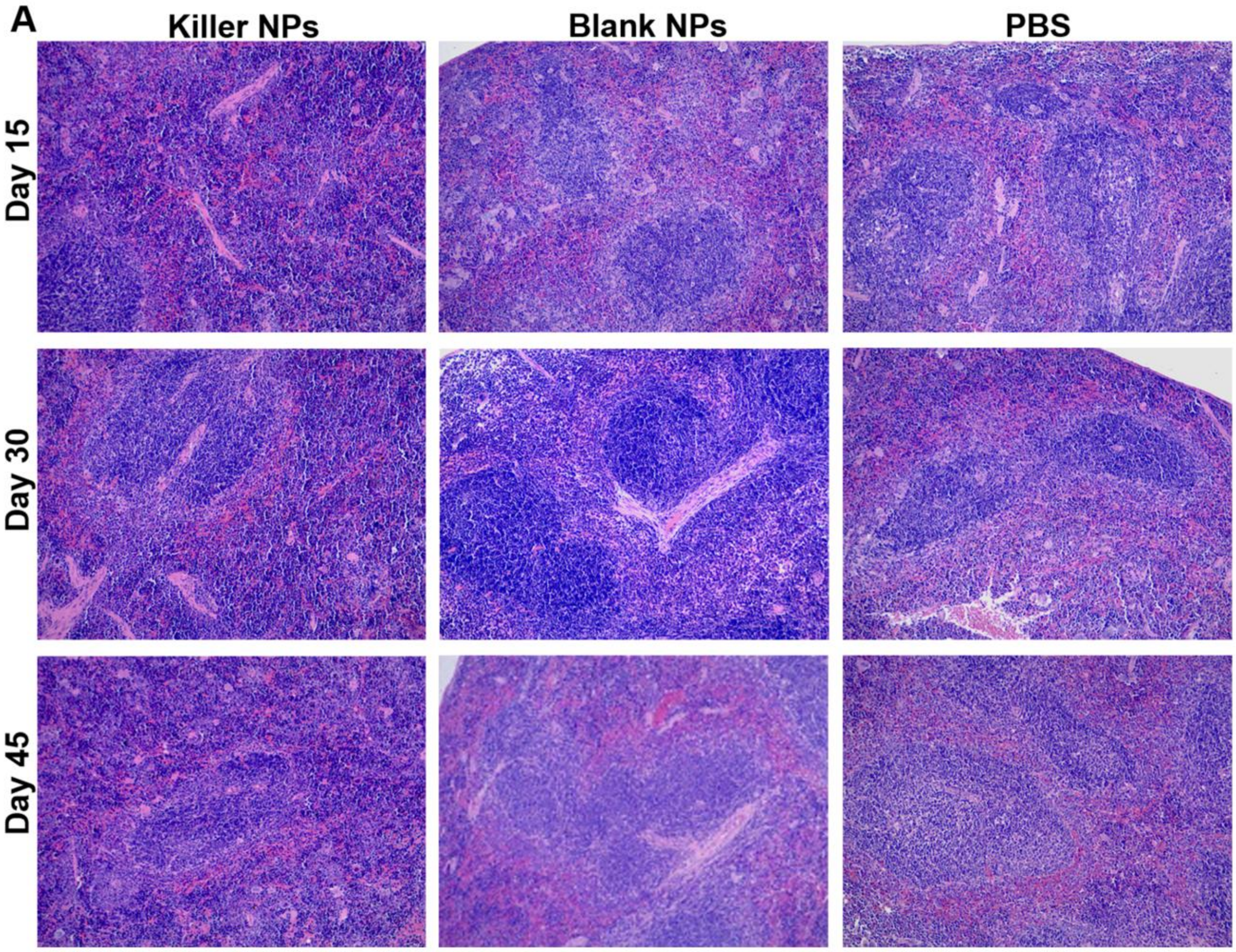

Fig. S7A No apparent pathological injuries were observed in the recipient vital organs after treatment with killer NPs. On days 15, 30 and 45 after skin transplantation (2, 17 and 32 days after the final injection of killer NPs, blank NPs, or PBS), spleen, kidney, liver, heart, and lungs were isolated from each recipient mouse in each group. The paraffin-embedded sections were prepared, routinely stained with H&E, and analyzed for pathological injuries. The representative images of H&E staining for spleen were presented at different time points in each group. n = 3 mice per group at each time point. No visible difference was found across groups.

Kidney

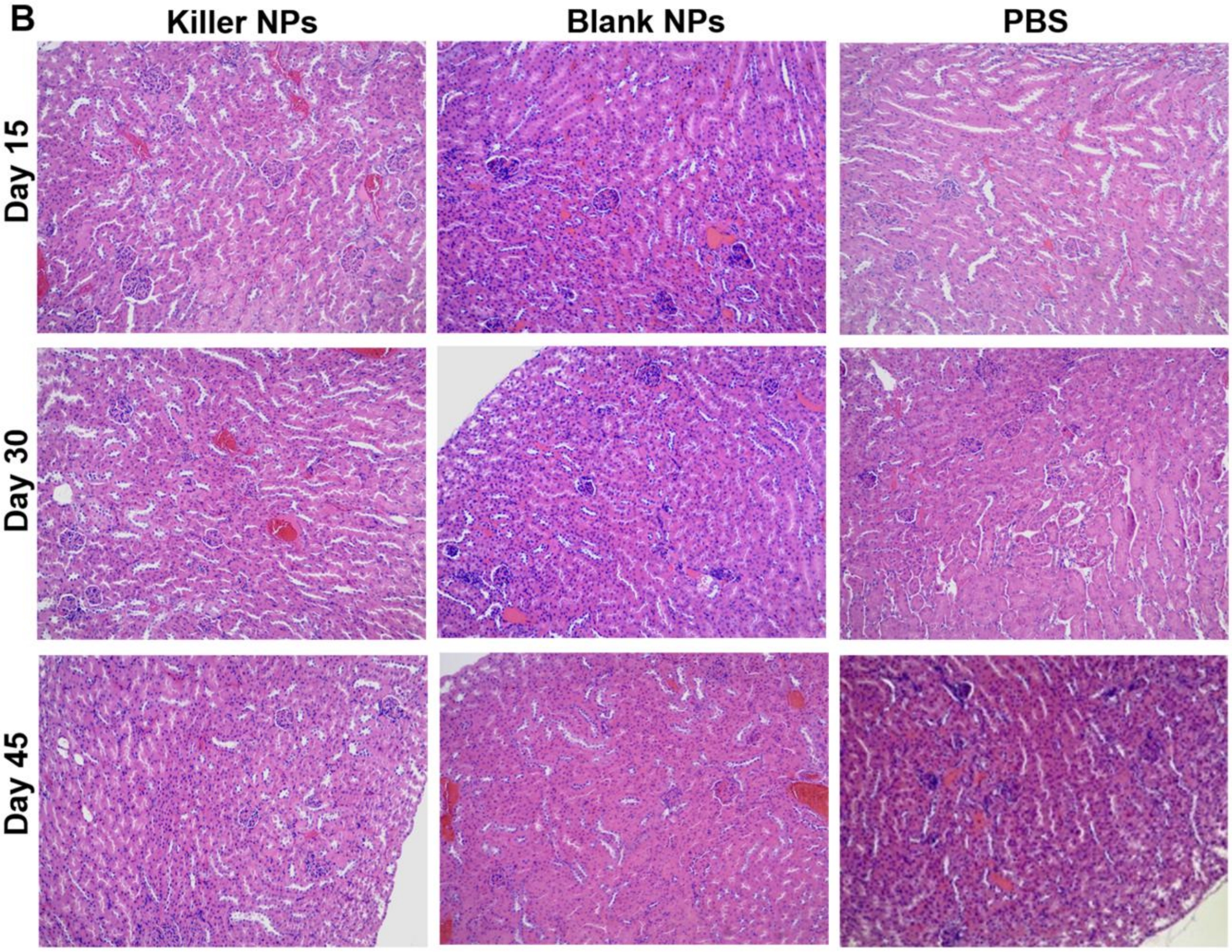

Fig. S7B No apparent pathological injuries were observed in the recipient vital organs after treatment with killer NPs. On days 15, 30 and 45 after skin transplantation (2, 17 and 32 days after the final injection of killer NPs, blank NPs, or PBS), spleen, kidney, liver, heart, and lungs were isolated from each recipient mouse in each group. The paraffin-embedded sections were prepared, routinely stained with H&E, and analyzed for pathological injuries. The representative images of H&E staining for kidney were presented at different time points in each group. n = 3 mice per group at each time point. No visible difference was found across groups.

Liver

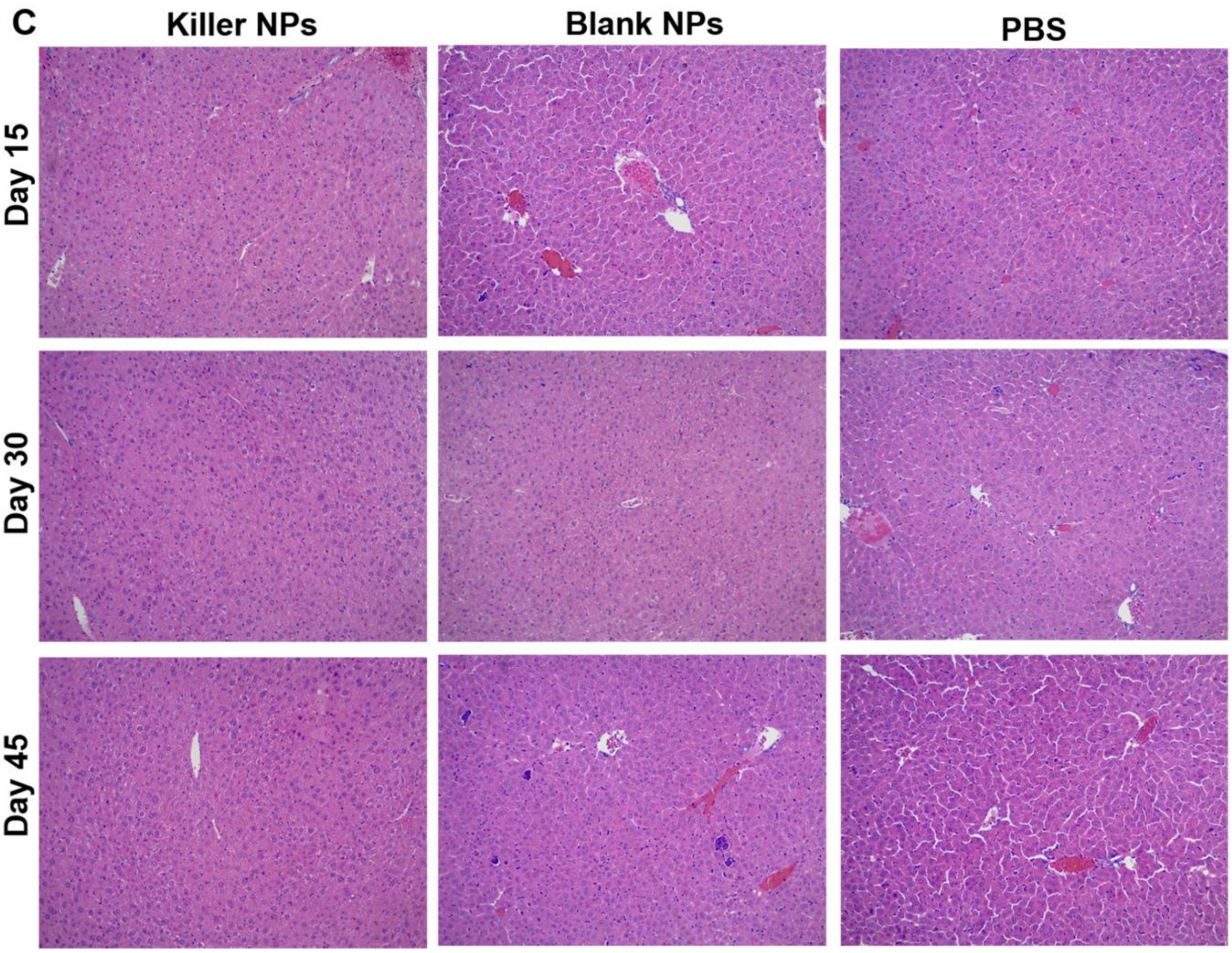

Fig. S7C No apparent pathological injuries were observed in the recipient vital organs after treatment with killer NPs. On days 15, 30 and 45 after skin transplantation (2, 17 and 32 days after the final injection of killer NPs, blank NPs, or PBS), spleen, kidney, liver, heart, and lungs were isolated from each recipient mouse in each group. The paraffin-embedded sections were prepared, routinely stained with H&E, and analyzed for pathological injuries. The representative images of H&E staining for liver were presented at different time points in each group. n = 3 mice per group at each time point. No visible difference was found across groups.

Heart

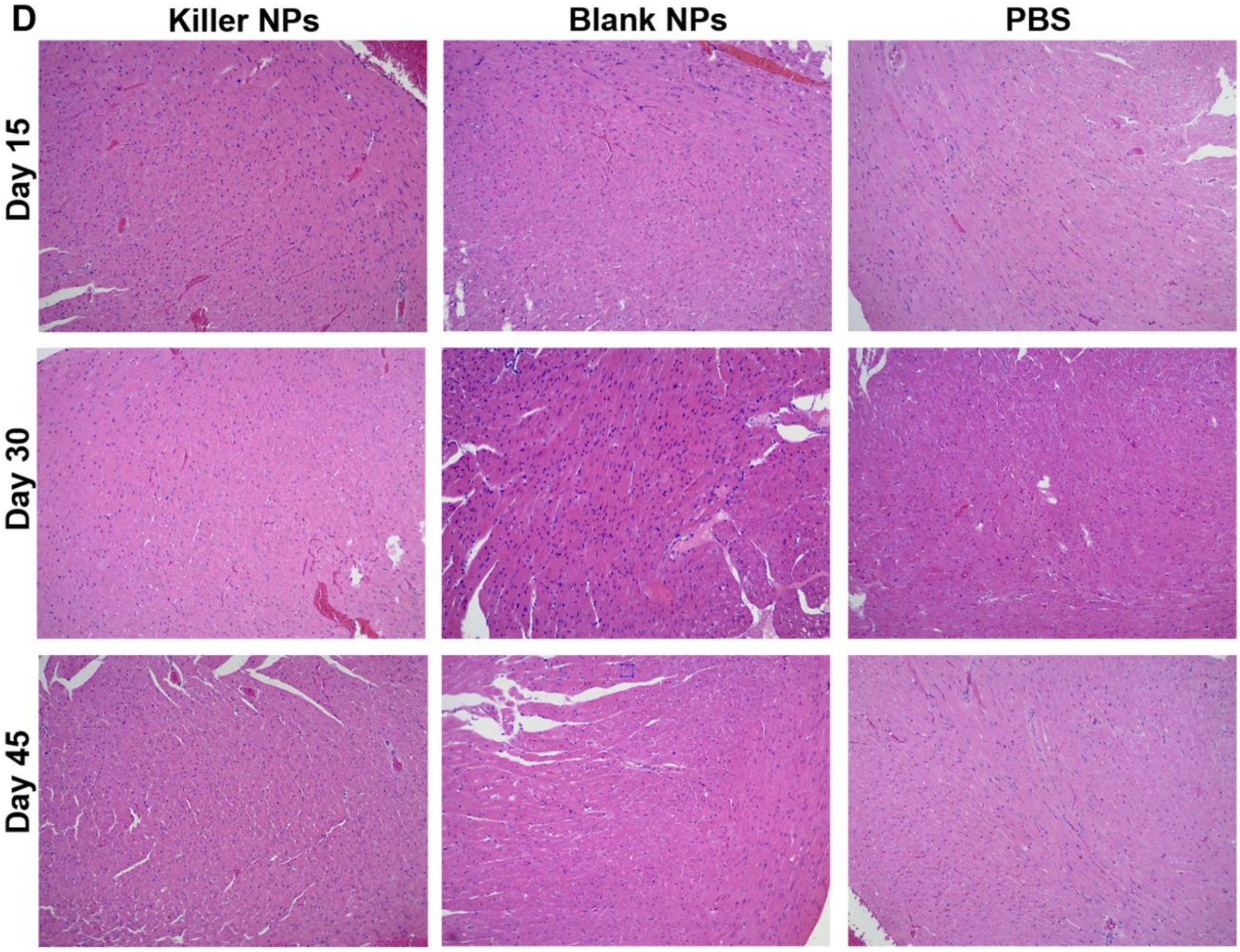

Fig. S7D No apparent pathological injuries were observed in the recipient vital organs after treatment with killer NPs. On days 15, 30 and 45 after skin transplantation (2, 17 and 32 days after the final injection of killer NPs, blank NPs, or PBS), spleen, kidney, liver, heart, and lungs were isolated from each recipient mouse in each group. The paraffin-embedded sections were prepared, routinely stained with H&E, and analyzed for pathological injuries. The representative images of H&E staining for heart were presented at different time points in each group. n = 3 mice per group at each time point. No visible difference was found across groups.

Lung

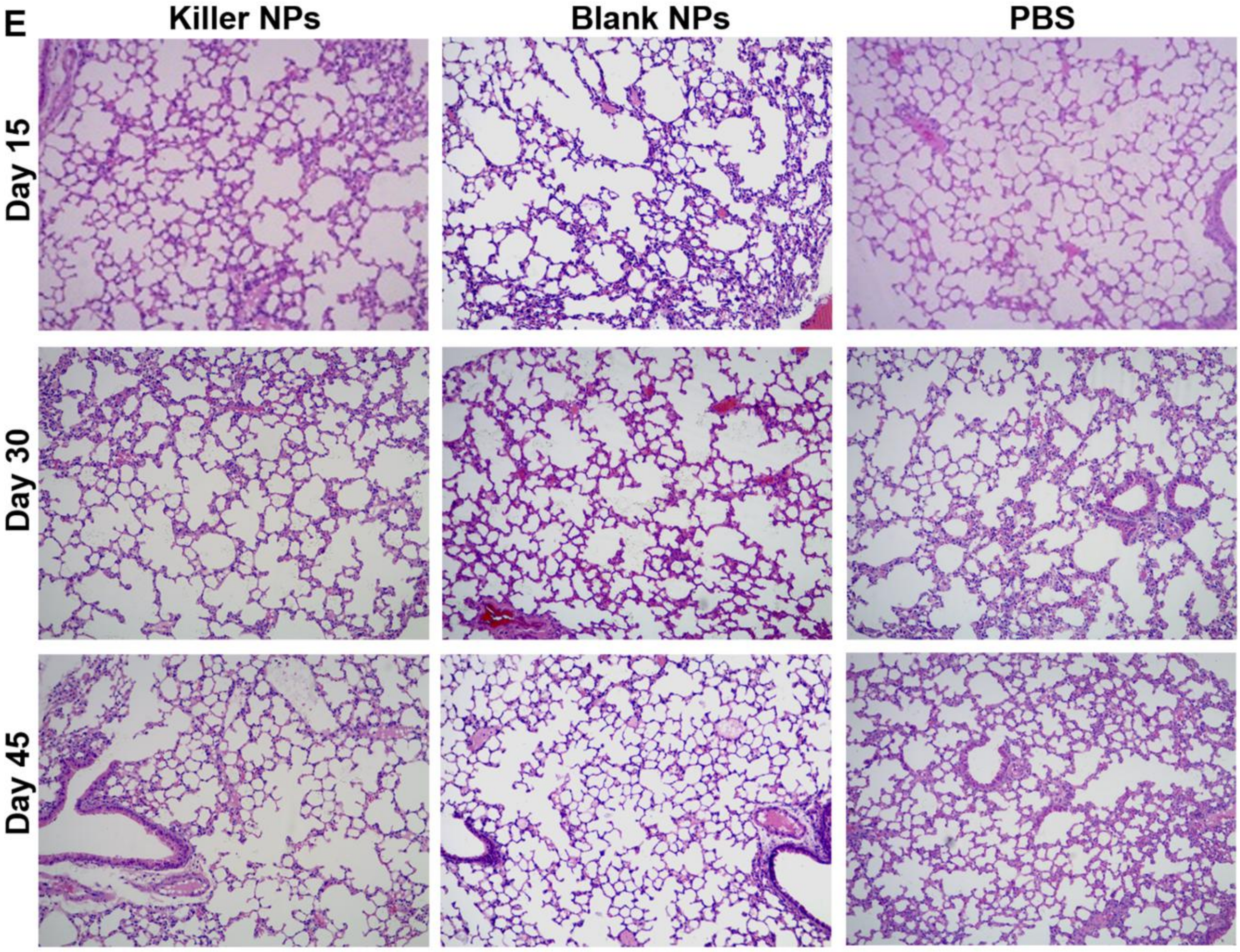

Fig. S7E No apparent pathological injuries were observed in the recipient vital organs after treatment with killer NPs. On days 15, 30 and 45 after skin transplantation (2, 17 and 32 days after the final injection of killer NPs, blank NPs, or PBS), spleen, kidney, liver, heart, and lungs were isolated from each recipient mouse in each group. The paraffin-embedded sections were prepared, routinely stained with H&E, and analyzed for pathological injuries. The representative images of H&E staining for lung were presented at different time points in each group. n = 3 mice per group at each time point. No visible difference was found across groups.
